# Apolipoprotein E Deficiency Impairs Human Microglial Proliferation Accompanied by Elevated Cellular Oxidative Stress

**DOI:** 10.1111/jcmm.71074

**Published:** 2026-03-20

**Authors:** Dayoung Kim, Takayuki Kondo, Keiko Imamura, Kayoko Tsukita, Ayako Nagahashi, Tomoki Sakasai, Haruhisa Inoue

**Affiliations:** ^1^ Center for iPS Cell Research and Application (CiRA) Kyoto University Kyoto Japan; ^2^ iPSC‐based Drug Discovery and Development Team RIKEN BioResourse Research Center (BRC) Kyoto Japan; ^3^ Medical‐risk Avoidance based on iPS Cells Team RIKEN Center for Advanced Intelligence Project (AIP) Kyoto Japan

**Keywords:** ApoE, cell cycle, inflammasome, iPSCs, lipid, microglia, oxidative stress, proliferation, TGF‐β signalling

## Abstract

The *APOE* gene, which encodes Apolipoprotein E (ApoE), is the strongest genetic risk locus for Alzheimer's disease (AD). A substantial fraction of AD risk genes converges on pathways controlling lipid metabolism and immune regulation, in which microglia serve as a central integrative hub in the brain. Although microglial phenotypes linked to different *APOE* genotypes have been extensively characterised, the fundamental question of how ApoE shapes the core functions of human microglia remains unresolved. Here, we generated *APOE* knockout (KO) microglia from AD patient–derived induced pluripotent stem cells (iPSCs) and characterised their cellular and molecular phenotypes. Ablation of *APOE* resulted in marked lipid droplet accumulation and increased NLRP3 inflammasome activation. Transcriptomic analysis further revealed downregulation of cell cycle–related pathways, accompanied by enrichment of an oxidative stress–associated pathway. Consistent with these transcriptomic signatures, *APOE* KO microglia exhibited elevated intracellular reactive oxygen species (ROS) levels and a marked reduction in proliferative capacity. Given the importance of microglial proliferation for maintaining immune homeostasis in the brain, our findings highlight ApoE as being an important regulator of this process, with potential consequences for the pathogenesis of neurodegenerative disorders.

## 1 Introduction

Alzheimer's disease (AD) is a progressive neurodegenerative disorder, with the majority of cases occurring sporadically through the combined influence of genetic and environmental factors [1, 2]. Genome‐wide association studies have shown that many AD risk loci converge on pathways involved in lipid metabolism and innate immune regulation [3, 4]. Among these, *APOE*, the gene encoding Apolipoprotein E (ApoE), represents the strongest genetic risk locus for AD [5]. ApoE functions as a lipid‐binding protein essential for lipid transport in the central nervous system, and accumulating evidence indicates that it also modulates innate immune responses, including those mediated by microglia [6].

Microglia, the resident macrophages of the brain, serve as a key cellular interface between lipid handling and innate immune responses. They maintain brain homeostasis through constant environmental surveillance, phagocytosis, lipid processing and inflammatory regulation [7, 8]. *APOE* genotype has been shown to modify microglial states, including cellular alterations in inflammatory signalling, lysosomal function and metabolic pathways [9, 10, 11, 12, 13, 14, 15, 16, 17]. Despite these insights, most prior work has focused on isoform‐specific differences or disease‐associated conditions, leaving the fundamental role of ApoE in human microglial physiology unresolved.

To address this gap, we generated *APOE* knockout (KO) microglia from AD patient–derived induced pluripotent stem cells (iPSCs) to directly assess the consequences of ApoE loss in human microglia. Our analyses revealed distinct alterations in cellular behaviour and transcriptional regulation, including previously unrecognised deficits in proliferative capacity. These findings provide new insight into the role of ApoE in maintaining human microglial homeostasis and may help refine our understanding of how microglial biology contributes to AD pathogenesis.

## 2 Materials and Methods

### 2.1 Generation and Culture of Human iPSCs

The *APOE* ε3/ε3 iPSC line was generated from human peripheral blood mononuclear cells (PBMCs) derived from a sporadic AD patient, as described previously [18]. Briefly, PBMCs were reprogrammed using episomal vectors encoding the human reprogramming factors SOX2, KLF4, OCT4, L‐MYC, LIN28 and dominant‐negative p53. Several days after transduction, the cells were collected and replated onto laminin (iMatrix‐511; Nippi, Tokyo, Japan)–coated dishes. The following day, the culture medium was replaced with StemFit AK03 (Ajinomoto, Tokyo, Japan), which was subsequently refreshed every other day. Approximately 20 days after transduction, emerging iPSC colonies were manually isolated and expanded.

iPSCs were maintained on laminin‐coated plates in StemFit AK02N medium (Ajinomoto) supplemented with penicillin (100 U/mL)–streptomycin (100 μg/mL) (Gibco, Thermo Fisher Scientific, Waltham, MA) at 37°C in a humidified 5% CO_2_ incubator. For passaging, iPSC colonies were dissociated into single cells using TrypLE Select (Gibco, Thermo Fisher Scientific) at 37°C. The resulting single‐cell suspensions were seeded in the StemFit medium containing 10 μM Y‐27632 ROCK inhibitor (Nacalai Tesque, Kyoto, Japan). On the following day, the medium was replaced with inhibitor‐free StemFit and subsequently refreshed every 2–3 days.

### 2.2 HMC3 Cell Culture

The HMC3 human microglial cell line (ATCC CRL‐3304; LGC Standards, Manassas, VA, USA) was obtained from the American Type Culture Collection. Cells were cultured in Eagle's Minimum Essential Medium (EMEM; ATCC) supplemented with 10% fetal bovine serum (FBS; Gibco, Thermo Fisher Scientific) and penicillin (100 U/mL)–streptomycin (100 μg/mL) (Gibco, Thermo Fisher Scientific) at 37°C in a humidified atmosphere with 5% CO_2_. Adherent cells were detached using 0.25% Trypsin (Gibco, Thermo Fisher Scientific), and the culture medium was replaced every 2–3 days.

### 2.3 Generation of *APOE* KO iPSCs

*APOE* KO iPSCs were generated using the Alt‐R CRISPR‐Cas9 System (Integrated DNA Technologies, Coralville, IA, USA). CRISPR RNA (crRNA, 5′‐GCGTTGCTGGTCACATTCC‐3′; Integrated DNA Technologies) targeting exon 2 of the *APOE* gene was duplexed with trans‐activating CRISPR RNA (tracrRNA, Integrated DNA Technologies) according to the manufacturer's protocol. The crRNA–tracrRNA complexes were co‐electroporated with Alt‐R *S.p*. Cas9‐GFP nuclease V3 (Integrated DNA Technologies) using a 4D‐Nucleofector system (Lonza, Basel, Switzerland) to induce double‐strand breaks followed by non‐homologous end joining (NHEJ). Resulting insertions and deletions at the targeted locus were confirmed by Sanger sequencing. For off‐target analysis, potential off‐target sites were predicted using Cas‐OFFinder [19], allowing up to two mismatches and a DNA bulge size ≤ 1. Of the predicted sites, loci for which suitable PCR primers could be designed (400–600 bp amplicons spanning the predicted Cas9 cut sites) were analysed by Sanger sequencing.

### 2.4 Generation of *APOE* KO HMC3

*APOE* KO HMC3 cells were established using the Alt‐R CRISPR‐Cas9 System (Integrated DNA Technologies). crRNA (5′‐GGTTCTGTGGGCTGCGTTGC‐3′; Integrated DNA Technologies) targeting exon 2 of the *APOE* gene was duplexed with tracrRNA–ATTO 550 (Integrated DNA Technologies) according to the manufacturer's protocol. The crRNA–tracrRNA complexes were co‐electroporated with Alt‐R *S.p*. Cas9‐GFP nuclease V3 (Integrated DNA Technologies) and a single‐stranded oligodeoxynucleotide (ssODN; 5′‐AGCAAGCCCCGCCCCCATACCTGCCAGGAATGTGACGAGCTACGCAGCCCACAGAACCTTCATCTTCCTGCCTGTGATTGG‐3′; Integrated DNA Technologies) using a 4D‐Nucleofector system (Lonza). Forty‐eight hours after nucleofection, ATTO 550^+^/GFP^+^ cells were subjected to single‐cell sorting by FACS (BD FACSAria II, BD Biosciences, San Jose, CA, USA). Individual clones were expanded, and genomic DNA was extracted from each clone for Sanger sequencing analysis.

### 2.5 Differentiation of iPSCs Into iMGLs

Induction of iMGLs was performed according to the protocol described by Brownjohn et al. [20], with minor modifications. iPSCs were seeded 4–7 days after the last passage at a density of 1.0 × 10^4^ cells per well into 96‐well ultra‐low‐attachment, round‐bottom plates (7007; Corning Incorporated, Corning, NY, USA) containing 100 μL of StemFit AK02N medium (Ajinomoto) supplemented with 10 μM Y‐27632 (Nacalai Tesque), 50 ng/mL BMP4 (Peprotech), 20 ng/mL SCF (Peprotech) and 50 ng/mL VEGF_121_ (Peprotech). Plates were centrifuged at 300 *g* for 3 min and cultured for 4 days, with a half‐medium change on day 2. One to two embryoid bodies (EBs) per 1 cm^2^ were transferred to tissue‐culture‐treated 6‐, 12‐, or 24‐well plates and cultured in X‐VIVO 15 medium (Lonza) supplemented with 2 mM GlutaMax (Gibco, Thermo Fisher Scientific), 0.5 mM monothioglycerol (FUJIFILM Wako Pure Chemical Corporation, Osaka, Japan), penicillin (100 U/mL)–streptomycin (100 μg/mL) (Gibco, Thermo Fisher Scientific), 100 ng/mL M‐CSF (Peprotech) and 25 ng/mL IL‐3 (Peprotech). Two‐thirds of the medium was replaced every 4–7 days. Emergent primitive macrophage precursors (PMPs) were harvested from the suspension on day 30 and plated at a density of 5.0 × 10^4^ cells/cm^2^ in RPMI 1640 medium (Gibco, Thermo Fisher Scientific). PMPs adhered to the uncoated surface within 1 h under serum‐free conditions, after which the medium was replaced with complete microglia maturation medium consisting of RPMI 1640 (Gibco, Thermo Fisher Scientific) supplemented with 10% FBS (Gibco, Thermo Fisher Scientific), 2 mM GlutaMAX (Gibco, Thermo Fisher Scientific), penicillin (100 U/mL)–streptomycin (100 μg/mL) (Gibco, Thermo Fisher Scientific), 100 ng/mL IL‐34 (Peprotech) and 10 ng/mL GM‐CSF (Peprotech). The medium was refreshed every 2–3 days, and on maturation day 8, iMGLs were collected for downstream analyses.

### 2.6 Immunocytochemistry

Cells were fixed with 4% paraformaldehyde (Nacalai Tesque) for 5–10 min at room temperature and washed three times with PBS (Nacalai Tesque). Samples were then blocked for 1 h in 0.1% Tween‐20 (Nacalai Tesque) in PBS (PBS‐T) containing 10% donkey serum (Sigma‐Aldrich, St. Louis, MO, USA) or 1% bovine serum albumin (Nacalai Tesque) and incubated overnight at 4°C with primary antibodies diluted in the same blocking solution. After three washes with PBS‐T, cells were incubated with Alexa Fluor–conjugated secondary antibodies (1:1000; Invitrogen, Thermo Fisher Scientific) for 1 h at room temperature in the dark. For intracellular immunostaining, cells were permeabilized with 0.1% Triton X‐100 (Nacalai Tesque) in PBS for 5 min before the blocking step. For iPSCs, permeabilization was performed using 0.3% Triton X‐100 under the same conditions. For nuclear staining, DAPI (4′,6‐diamidino‐2‐phenylindole; Invitrogen, Thermo Fisher Scientific) was used. Fluorescence images were obtained using an FV3000 confocal microscope (Olympus, Tokyo, Japan), and image analysis was performed with ImageJ software (NIH, Bethesda, MD, USA). Primary antibodies used in this study included anti‐TREM2 (1:100; MAB17291; R&D Systems, Minneapolis, MN, USA), anti‐IBA1 (1:100; ab5076; Abcam, Cambridge, UK), anti‐P2RY12 (1:200; HPA014518; Sigma‐Aldrich), anti‐Ki‐67 (1:100; M7240; Agilent Technologies, Santa Clara, CA, USA), anti–phospho‐histone H3 (Ser10) (1:200; 9701; Cell Signalling Technology, Danvers, MA, USA), anti‐CD43 (1:100; ab235453; Abcam), anti‐CD43 (1:25; 14‐0439‐82; eBioscience, San Diego, CA, USA), anti‐NANOG (1:100; AF1997; R&D Systems), anti‐NANOG (1:800; 3580; Cell Signaling Technology) and anti‐SSEA‐4 (1:1000; MAB4304; Chemicon, Temecula, CA, USA).

### 2.7 Lipid Staining

Following immunocytochemistry, cells were washed three times with PBS and incubated with BODIPY 493/503 (2 μM; Cayman Chemical, Ann Arbor, MI, USA) diluted in PBS for 30 min at room temperature in the dark. After incubation, cells were gently rinsed with PBS prior to imaging.

### 2.8 EdU Incorporation Assay

Cell proliferation was assessed using the Click‐it EdU Alexa Fluor 647 detection system (Invitrogen, Thermo Fisher Scientific) according to the manufacturer's instructions. Briefly, iMGLs were incubated with 5‐ethynyl‐2′‐deoxyuridine (EdU) for 24 h, whereas iPSCs and PMPs were labelled with EdU for 1 h. Following EdU incorporation, cells were fixed with 4% paraformaldehyde (Nacalai Tesque) and subjected to immunofluorescence staining. Incorporated EdU was subsequently detected via a copper‐catalysed azide–alkyne cycloaddition reaction using Alexa Fluor 647–conjugated azide.

### 2.9 Oxidative Stress Detection

Intracellular reactive oxygen species (ROS) were assessed using the CellROX Green reagent (Thermo Fisher Scientific), according to the manufacturer's instructions. Briefly, iMGLs were incubated with CellROX Green (5 μM) diluted in culture medium for 30 min at 37°C, followed by gentle washing with PBS. Cells were then fixed with 4% paraformaldehyde (Nacalai Tesque) for 15 min at room temperature and processed for subsequent immunofluorescence staining as described above. CellROX Green fluorescence was acquired using standard excitation/emission settings (485/520 nm) on an FV3000 confocal microscope (Olympus) within 24 h after fixation. For visualisation, CellROX Green signals are displayed in orange pseudocolor. Quantitative image analysis was performed using ImageJ software (NIH). As a positive control, cells were treated with tert‐butyl hydroperoxide (200 μM; FUJIFILM Wako Pure Chemical Corporation) for 30 min prior to CellROX Green incubation.

### 2.10 Quantitative Real‐Time PCR (qPCR) Analysis

Using the miRNeasy Mini Kit (Qiagen, Hilden, Germany), total RNA was extracted according to the manufacturer's instructions. For complementary DNA synthesis, 900 ng of total RNA was reverse‐transcribed with ReverTra Ace ‐α‐ (Toyobo, Osaka, Japan). Quantitative real‐time PCR analysis was then performed on the StepOnePlus Real‐Time PCR System (Applied Biosystems; Thermo Fisher Scientific) using TB Green Premix Ex Taq II (Tli RNaseH Plus) (Takara Bio, Shiga, Japan). The primer sequences used were as follows: *ACTB* forward, 5′‐CACAGAGCCTCGCCTTT‐3′; *ACTB* reverse, 5′‐GAGCGCGGCGATATCAT‐3′; *TMEM119* forward, 5′‐AGTCCTGTACGCCAAGGAAC‐3′; *TMEM119* reverse, 5′‐GCAGCAACAGAAGGATGAGG‐3′; *C1QA* forward, 5′‐GGGAAGAAAGGGGAGGCAGG‐3′; *C1QA* reverse, 5′‐GTTTCCAGAGGGCCCAGGTT‐3′; *PROS1* forward, 5′‐AAGAAGCCAGGGAGGTCTTTG‐3′; *PROS1* reverse, 5′‐ACGTGCAGCAGTGAATAACC‐3′; *HEXB* forward, 5′‐GGGAGCATTACGAGGTTTAGAG‐3′; *HEXB* reverse, 5′‐GGTGGATTCATTGATGGTGAAAG‐3′; *GPR34* forward, 5′‐CTCCCACAGAATGCGCTTTAT‐3′; *GPR34* reverse, 5′‐AGAGGGCGATTATGTTCCCAA‐3′; *SMAD7* forward, 5′‐TTCCTCCGCTGAAACAGGG‐3′; *SMAD7* reverse, 5′‐CCTCCCAGTATGCCACCAC‐3′; *ID3* forward, 5′‐GAGAGGCACTCAGCTTAGCC‐3′; *ID3* reverse, 5′‐TCCTTTTGTCGTTGGAGATGAC‐3′.

### 2.11 Sanger Sequencing

Genomic DNA was extracted using the PureLink Genomic DNA Mini Kit (Invitrogen, Thermo Fisher Scientific). The genomic region containing the target locus was amplified by PCR with PrimeSTAR Max DNA Polymerase (Takara Bio) using the following primer pairs: *APOE* KO forward, 5′‐ATGCAACAAGGCTTGGAAGGCTAACCTGG‐3′; *APOE* KO reverse, 5′‐ATAAAGATCACAGCTGCCCCGTGTCTGG‐3′; off‐target site 1 (OT1) forward, 5′‐GTCTTAGTGGCTCTAGAGGATGT‐3′; OT1 reverse, 5′‐GAGAAAGAAATGGAGTCCGAGC‐3′; OT2 forward, 5′‐AAGGCCTTCTGCTGGAATCA‐3′; OT2 reverse, 5′‐GTCTGACAAGCAAGGCCAGA‐3′; OT4 forward, 5′‐AGTGCCAGGAGATCTAGGCCA‐3′; OT4 reverse, 5′‐AGTCAACAGTGAGCGGATCCAA‐3′. The resulting PCR products were purified with the QIAquick Gel Extraction Kit (Qiagen) and subjected to Sanger sequencing using the BigDye Terminator v3.1 Cycle Sequencing Kit (Applied Biosystems; Thermo Fisher Scientific). Sequencing reactions were cleaned up using the BigDye XTerminator Purification Kit (Applied Biosystems; Thermo Fisher Scientific) and subsequently analysed on a 3500 xL Genetic Analyser (Applied Biosystems; Thermo Fisher Scientific). Resulting chromatograms were visualised and aligned in SnapGene software (GSL Biotech LLC, Chicago, IL, USA).

### 2.12 Capillary Western Blot (Wes)

Capillary western blot analyses were performed using the Wes system (ProteinSimple, San Jose, CA, USA) according to the manufacturer's instructions. Cell lysates were prepared using 1× RIPA buffer (Nacalai Tesque) supplemented with cOmplete Mini protease inhibitor cocktail (Roche Diagnostics GmbH, Mannheim, Germany) and PhosSTOP phosphatase inhibitor (Roche Diagnostics GmbH). Lysates were diluted with 0.1× sample buffer and denatured at 95°C for 5 min prior to loading. The following primary antibodies were used: Anti‐ApoE (1:20; AF4144; R&D Systems) and anti‐β‐actin (1:400; A5441; Sigma‐Aldrich). In this system, proteins are separated by capillary electrophoresis, and molecular weights are reported as apparent values relative to internal size standards. As a result, apparent molecular weight estimates may differ from those obtained by SDS–PAGE [21].

### 2.13 Phagocytosis Assay

The phagocytosis assay was performed with slight modifications from the method described by Brownjohn et al. [20]. On day 7 of maturation, the culture medium of iMGLs was replaced with complete microglia maturation medium prepared using phenol‐red free RPMI 1640 (Gibco, Thermo Fisher Scientific) supplemented with 1% FBS (Gibco, Thermo Fisher Scientific). On the following day (day 8), cells were incubated with *Staphylococcus aureus* ( *S. aureus* ) bioparticles conjugated with pHrodo Red (Al0010; Invitrogen, Thermo Fisher Scientific) and briefly centrifuged. The plates were immediately transferred to the Incucyte ZOOM live‐cell imaging system (Sartorius, Göttingen, Germany), and phase‐contrast and red fluorescence images were acquired every 15 min for 3 h. As a positive control for reduced phagocytic activity, cells were pretreated with 10 μM cytochalasin D (Cayman Chemical) for 45 min and co‐incubated throughout the imaging.

### 2.14 IL‐1β Measurement

iMGLs were primed with lipopolysaccharide (LPS O55:B5, 100 ng/mL; Sigma‐Aldrich) for 4 h and subsequently activated with ATP (5 mM; InvivoGen, San Diego, CA, USA) for 30 min to trigger canonical NLRP3 inflammasome signalling and IL‐1β release. For the inhibitory control of NLRP3 activation, MCC950 (1 μM; InvivoGen) was added 30 min prior to ATP stimulation. Cell‐free supernatants were collected 30 min after ATP treatment for LPS + ATP and MCC950 conditions, or after 4 h of LPS stimulation alone.

IL‐1β concentrations in supernatants were quantified using the AlphaLISA Human Interleukin 1β Detection Kit (Revvity, formerly PerkinElmer, Waltham, MA, USA) following the manufacturer's instructions. Briefly, standards and samples were dispensed into 384‐well plates, incubated with acceptor beads and biotinylated antibody, followed by streptavidin donor beads, and the signal was measured using an EnVision 2104 Multilabel Reader (Revvity). Concentrations were calculated from a 4‐parameter logistic (4PL) standard curve fitted by nonlinear regression and 1/*Y* ^2^ weighting, as recommended by the manufacturer.

### 2.15 HMC3 Proliferation Assay

HMC3 cells were seeded in 96‐well plates (Thermo Fisher Scientific) at a density of 1.6 × 10^4^ cells per well in EMEM (ATCC) containing 10% FBS (Gibco, Thermo Fisher Scientific) and penicillin (100 U/mL)–streptomycin (100 μg/mL) (Gibco, Thermo Fisher Scientific). The following day, plates were placed in the Incucyte ZOOM system (Sartorius) for live cell imaging and phase‐contrast images were collected every 4 h over 5 days. The culture medium was refreshed immediately prior to imaging initiation (day 0) and again on day 2.

### 2.16 Bulk RNA Sequencing Analysis

Total RNA was extracted using the miRNeasy Mini Kit (Qiagen). RNA quality was assessed using the High Sensitivity RNA ScreenTape Assay on an Agilent 4200 TapeStation System (Agilent Technologies, Santa Clara, CA, USA). Samples that met the following quality criteria were used for library preparation: total RNA ≥ 1 ng, concentration ≥ 0.263 ng/μL, and RNA integrity number equivalent (RINe) ≥ 7. Full‐length cDNA was synthesised and amplified using the SMART‐Seq mRNA Kit (Takara Bio). Sequencing libraries were prepared with the Nextera XT DNA Library Preparation Kit (Illumina, San Diego, CA, USA) and indexed using the IDT for Illumina–DNA/RNA UD Indexes (Tagmentation) (Integrated DNA Technologies). Indexed libraries were sequenced on a NovaSeq X Plus platform (Illumina) using the NovaSeq X Series 25B Reagent Kit to generate paired‐end 150 bp reads. Base calling, demultiplexing, and alignment were performed using the DRAGEN Bio‐IT Platform v4.3.6 (Illumina). Sequencing reads were aligned to the human reference genome (GRCh38, GENCODE release 39).

RNA sequencing analysis was performed in R (v4.4.2). For visualisation, raw count data were transformed using the variance‐stabilising transformation (VST) in DESeq2, followed by removal of known batch effects with limma::removeBatchEffect, while preserving genotype‐associated variation. The resulting batch‐corrected expression matrix was used to generate Spearman correlation heatmaps and principal component analysis (PCA) plots to confirm appropriate sample clustering by biological group. For differential expression analysis, genes were filtered in two steps: (1) lowly expressed genes with fewer than 10 counts were removed and (2) genes showing excessive within‐group variability (top 10% standard deviation based on VST‐transformed data) were excluded. To account for hidden sources of variation, surrogate variable analysis (SVA) was applied [22]. Differentially expressed genes (DEGs) were identified using DESeq2 with a model incorporating both known batch and surrogate variables (design = ~ batch + SVs + genotype). The *APOE* ^+/+^ genotype was used as the reference group, and log_2_ fold changes (LFC) were shrunk using the apeglm method for stable estimation. Genes were considered to be significantly differentially expressed at adjusted *p* < 0.1 and |LFC| > 0.263. Gene set enrichment analysis (GSEA) was performed using the fgsea package (v1.32.0) on genes ranked by the DESeq2 Wald statistic. Pathways with adjusted *p* ≤ 0.05 and |normalised enrichment score (NES)| ≥ 1.3 were considered significantly enriched after Benjamini–Hochberg correction.

For canonical pathway and upstream regulator analyses conducted with Ingenuity Pathway Analysis (IPA; Qiagen), DEGs (adjusted *p* < 0.1, |LFC| > 0.263) from the *APOE* ^−/−^ vs. *APOE* ^+/+^ comparison were uploaded to the IPA platform. For upstream regulator analysis, predicted activation states were determined based on activation *z*‐scores and Fisher's exact test–derived overlap *p*‐values, with |*z*‐score| ≥ 2 and *p* < 0.05 regarded as significant. For Canonical Pathway Analysis, DEG‐based over‐represented pathways were identified in the IPA Knowledge Base using Fisher's exact test, and pathways with *p* < 0.05 were considered significantly enriched.

### 2.17 Statistical Analysis

All statistical analyses, except for bulk RNA sequencing, were performed using GraphPad Prism (v10.1.1; GraphPad Software, San Diego, CA, USA). Comparisons between two groups were evaluated using an unpaired two‐tailed Student's *t*‐test, while multiple group comparisons were evaluated by one‐way or two‐way ANOVA followed by the appropriate post hoc test (Tukey's or Šidák's multiple comparisons test, as indicated). Data are presented as mean ± SEM, and statistical significance was defined as *p* < 0.05.

## 3 Results

### 3.1 Generation of iMGLs Using Isogenic *APOE* ^+/+^ and *APOE* ^−/−^ iPSCs

**FIGURE 1:**
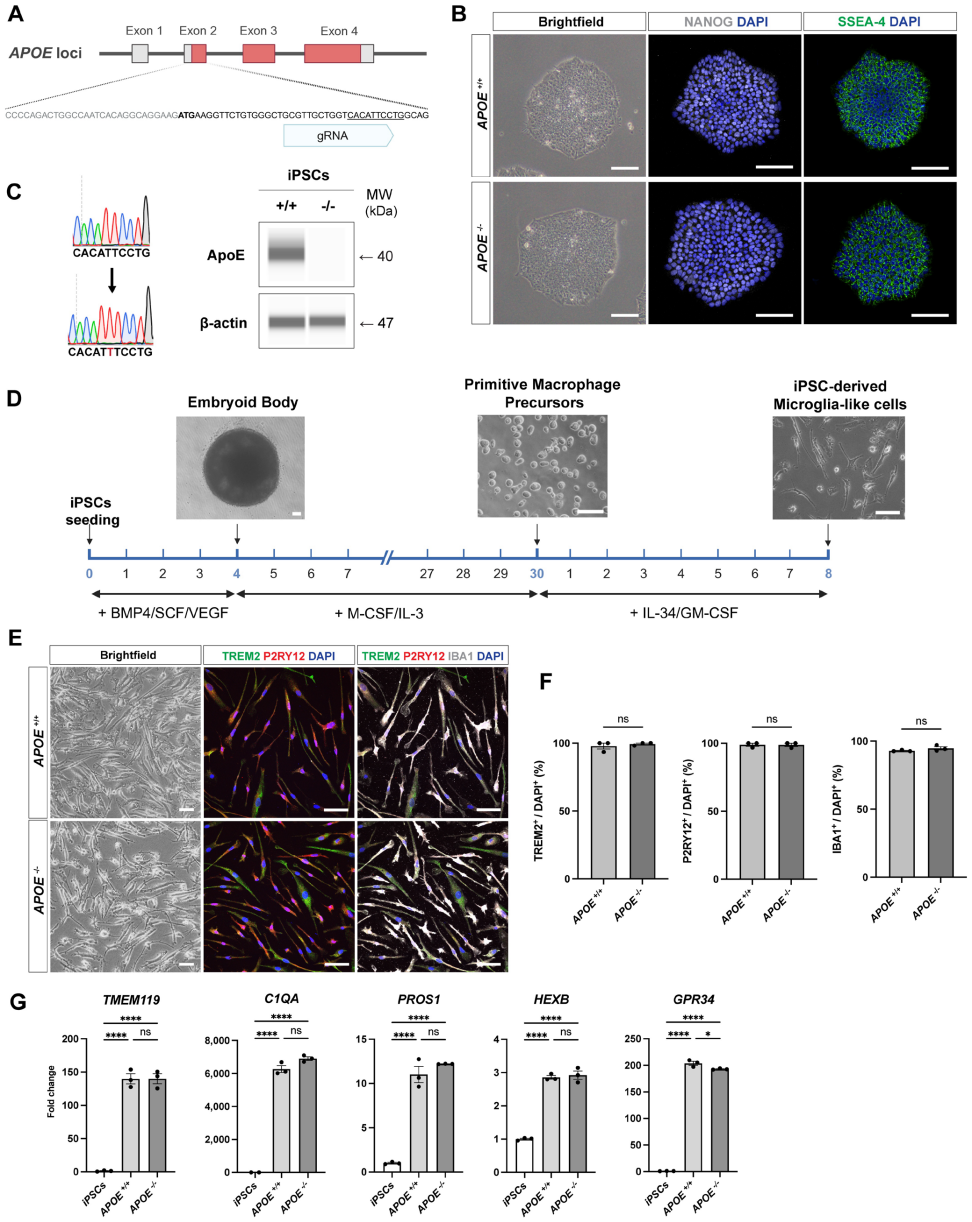
Generation of iPSC‐derived microglia‐like cells (iMGLs) from isogenic *APOE* ^+/+^ and *APOE* ^−/−^ iPSCs. (A) Schematic illustration of CRISPR‐Cas9 target sites used to generate *APOE* knockout (KO; *APOE* ^−/−^) iPSCs. Exon 2 sequences are shown below, with the guide RNA (gRNA) targeting site indicated. The underlined region corresponds to the edited sequence shown in panel C, which was confirmed by Sanger sequencing. (B) Brightfield image and immunostaining for the pluripotency markers NANOG (grey) and SSEA‐4 (green). Scale bars, 100 μm. (C) Validation of *APOE* KO by Sanger sequencing showing a frameshift insertion, and by capillary western blot analysis. (D) Overview of the differentiation timeline from iPSCs to iMGLs. Scale bars, 100 μm. (E) Representative brightfield and immunofluorescence images of iMGLs expressing microglia‐specific markers TREM2 (green), P2RY12 (red) and IBA1 (grey). Scale bars, 50 μm. (F) Quantification of marker‐positive cells (*n* = 3 wells per genotype). Statistical significance was determined using an unpaired two‐tailed Student's *t*‐test. ns, not significant. (G) Relative mRNA expression levels of microglial signature genes assessed by qPCR (*n* = 3 per genotype). Statistical analysis was performed using one‐way ANOVA followed by Tukey's post hoc test (*p < 0.05; ****p < 0.0001; ns, not significant).

To investigate the phenotype of human microglia under ApoE deficiency, we established an isogenic *APOE* KO iPSC line using CRISPR‐Cas9 genome editing (Figure 1A). The parental iPSC line carrying the *APOE* ε3/ε3 genotype, which represents the most prevalent isoform, was previously validated as exhibiting a normal karyotype [18] and will hereafter be referred to as *APOE* ^+/+^. Out of 24 single‐cell–derived clones screened, 9 clones (37.5%) harboured indel mutations at the target locus, among which 2 clones (8.3%) showed biallelic frameshift mutations. Following gene editing, the resulting *APOE* ^−/−^ iPSCs maintained the typical colony morphology of pluripotent stem cells and expressed canonical pluripotency markers including NANOG and SSEA‐4, as confirmed by immunocytochemistry (Figure 1B). Sanger sequencing verified the successful introduction of a frameshift insertion at exon 2 of the *APOE* locus, and capillary western blot analysis demonstrated the complete loss of ApoE protein, confirming biallelic disruption (Figure 1C). Analysis of selected predicted off‐target loci by Sanger sequencing did not detect insertions or deletions at the examined sites (Figure S1A,B).

Both *APOE* ^+/+^ and *APOE* ^−/−^ iPSCs were subsequently differentiated into iMGLs following a stepwise differentiation protocol based on previously established methods (Figure 1D) [20]. The resulting iMGLs displayed ramified morphology and expressed microglia‐specific markers such as TREM2, P2RY12 and IBA1, as shown by immunostaining (Figure 1E). Importantly, quantitative analysis showed that more than 90% of the cells were marker‐positive across both genotypes, with no significant difference between *APOE* ^+/+^ and *APOE* ^−/−^, indicating high culture purity in our preparations (Figure 1F). qPCR further confirmed robust and comparable expression of microglial signature genes [23], including *TMEM119*, *C1QA*, *PROS1* and *HEXB* between *APOE* ^+/+^ and *APOE* ^−/−^ iMGLs, thereby validating their microglial identity (Figure 1G). Together, these data demonstrate a well‐characterised isogenic iMGL model suitable for dissecting the mechanistic contributions of ApoE to microglial function.

### 3.2 Cellular Phenotypes Associated With ApoE Deficiency in iMGLs

**FIGURE 2:**
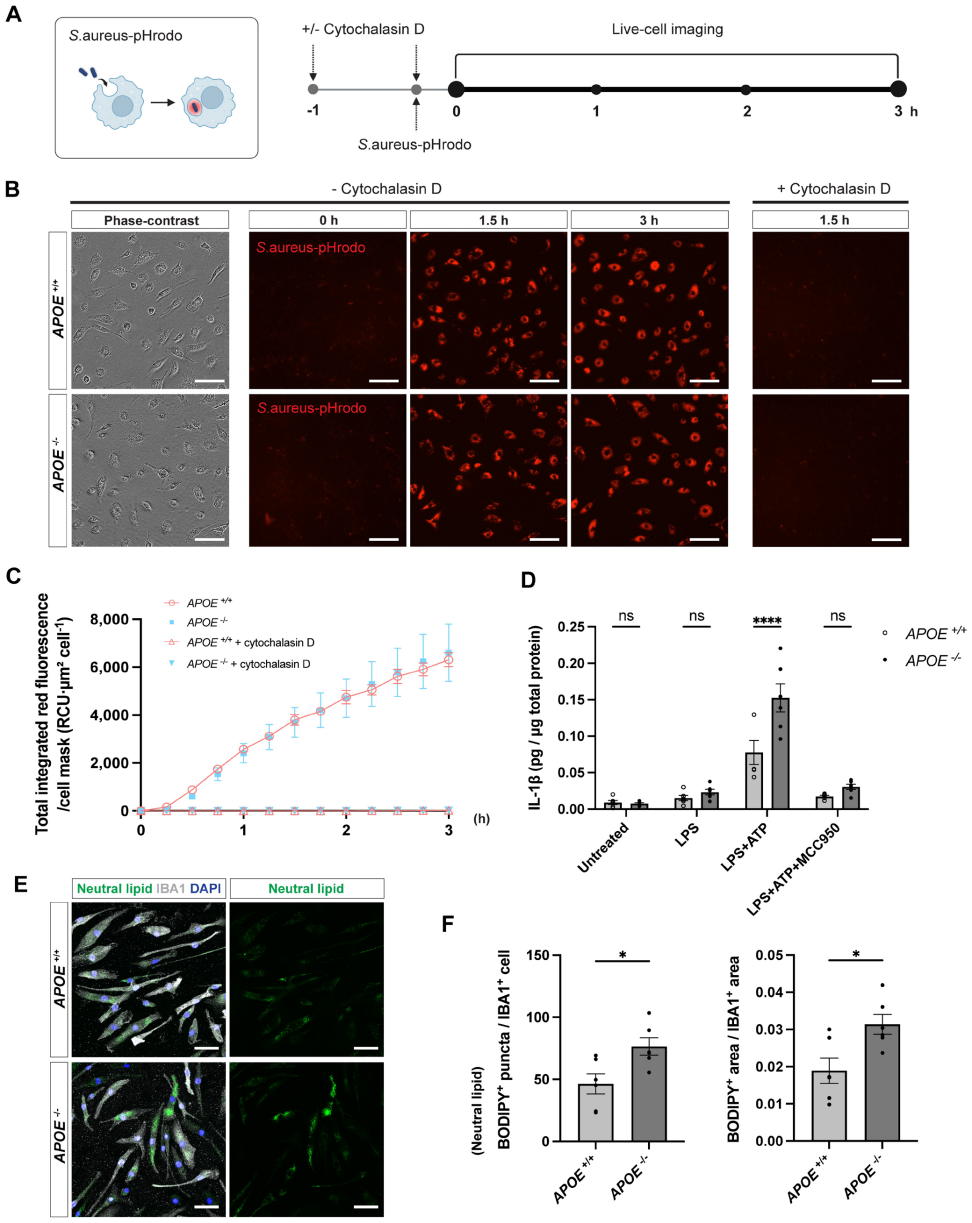
Cellular phenotypes associated with ApoE deficiency in iMGLs. (A) Schematic illustration of the *S. aureus*–pHrodo mechanism, which fluoresces red within acidic compartments, and the experimental timeline of live‐cell imaging for phagocytosis assay procedure. Phase‐contrast and red fluorescence images were acquired at 15‐min intervals. (B) Representative phase‐contrast and fluorescence images of iMGLs incubated with *S. aureus*–pHrodo bioparticles (red) in the presence or absence of 10 μM cytochalasin D (phagocytosis inhibitor). Scale bars, 100 μm. (C) Time‐course analysis of total integrated red fluorescence per cell mask (RCU·μm^2^ cell^−1^) over 3 h, showing comparable phagocytic kinetics between *APOE* ^+/+^ and *APOE* ^−/−^ iMGLs (*n* = 12 fields; four fields from three wells per genotype and condition). (D) IL‐1β levels in culture supernatants from iMGLs under the indicated stimulation conditions. Cells were primed with LPS (100 ng/mL, 4 h) and stimulated with ATP (5 mM, 30 min); MCC950 (1 μM) was added 30 min prior to ATP to inhibit NLRP3 inflammasome activation. IL‐1β concentrations were normalised to the total protein content of the corresponding cell lysates from each well (*n* = 6; two technical replicates from three wells per genotype). Statistical analysis was performed using a two‐way ANOVA followed by Šídák's multiple comparisons test. A significant genotype effect was observed only under the LPS + ATP condition (****p < 0.0001). (E) Representative fluorescence images of BODIPY (green), which labels neutral lipids, together with IBA1 (grey), a microglial marker. Scale bars, 50 μm. (F) Quantification of lipid droplet accumulation, represented as the number of BODIPY^+^ puncta per IBA1^+^ cell and the BODIPY^+^ area normalised to the IBA1^+^ area (*n* = 6 fields; two fields from three wells per genotype). Statistical significance was assessed using an unpaired two‐tailed Student's *t*‐test (*p < 0.05).

To assess functional consequences of ApoE loss, we first examined its effect on the phagocytic capacity of iMGLs. Live‐cell imaging was performed using *S. aureus* conjugated with pHrodo Red, a pH‐sensitive dye that fluoresces upon internalisation into acidic phagosomes (Figure 2A). Both *APOE* ^+/+^ and *APOE* ^−/−^ iMGLs readily engulfed *S. aureus* particles, exhibiting a time‐dependent increase in red fluorescence over the 3 h imaging period (Figure 2B). Quantitative analysis of total integrated red fluorescence per cell mask revealed comparable uptake kinetics between genotypes, indicating that the absence of ApoE does not overtly impair phagocytic activity under these conditions (Figure 2C). Treatment with 10 μM cytochalasin D, an actin polymerisation inhibitor, effectively abolished fluorescence in both groups, confirming that the observed signal originated from actin‐dependent phagocytic uptake (Figure 2B,C). This result is consistent with a recent report by Murphy et al. [14], which demonstrated comparable myelin and *E. coli* phagocytosis across *APOE* ε3 and KO microglia.

Given that microglia act as key innate immune effectors in the brain, we next asked whether ApoE deficiency alters their inflammatory responsiveness. Among the major signalling pathways orchestrating the innate immune response, the NLRP3 inflammasome represents one of the key mechanisms implicated across multiple neurological diseases [24]. We therefore examined whether ApoE loss affects NLRP3 inflammasome activation in human iMGLs. Following LPS priming and subsequent stimulation with ATP, a well‐established two‐step paradigm for NLRP3 inflammasome activation [25], *APOE* ^−/−^ iMGLs exhibited increased secretion of IL‐1β compared with *APOE* ^+/+^ controls (Figure 2D). While LPS priming alone did not produce a significant difference, subsequent stimulation with ATP, a canonical activator that induces K^+^ efflux [26], markedly increased IL‐1β release. This increase was attenuated by MCC950, a selective inhibitor of the NLRP3 inflammasome [27], indicating that the elevated IL‐1β production in *APOE* ^−/−^ iMGLs arises from enhanced NLRP3 inflammasome activation.

Given that ApoE plays a central role in lipid transport and cholesterol efflux, we hypothesised that ApoE deficiency would perturb lipid homeostasis in microglia. Consistent with this, previous studies have shown markedly elevated levels of cholesteryl ester species in *Apoe* ^−/−^ mouse microglia [28]. To assess lipid accumulation in our human model, we performed Boron‐Dipyrromethene (BODIPY) staining to visualise lipid droplets. Fluorescence microscopy revealed a marked increase in BODIPY‐positive puncta in *APOE* ^−/−^ iMGLs compared with *APOE* ^+/+^ controls (Figure 2E). Quantitative image analysis confirmed a significant increase in both lipid‐droplet number and total droplet area, indicating enhanced lipid accumulation (Figure 2F). These results demonstrate that the loss of ApoE leads to pronounced lipid droplet accumulation in human iMGL models.

Collectively, these results demonstrate that ApoE deficiency does not overtly impair phagocytosis but that it does promote NLRP3 inflammasome activation and robust lipid droplet accumulation, indicating a shift towards a pro‐inflammatory and metabolically altered state in human microglia.

### 3.3 Modest Global Transcriptomic Differences Between *APOE* ^+/+^ and *APOE* ^−/−^ iMGLs

To characterise transcriptomic changes associated with ApoE deficiency, bulk RNA sequencing was performed on iMGLs derived from parental and *APOE* KO iPSC lines. Because samples tended to cluster in batches (Figure S2A), we performed batch effect correction. Subsequent analyses were conducted using the batch‐corrected expression matrix.

**FIGURE 3:**
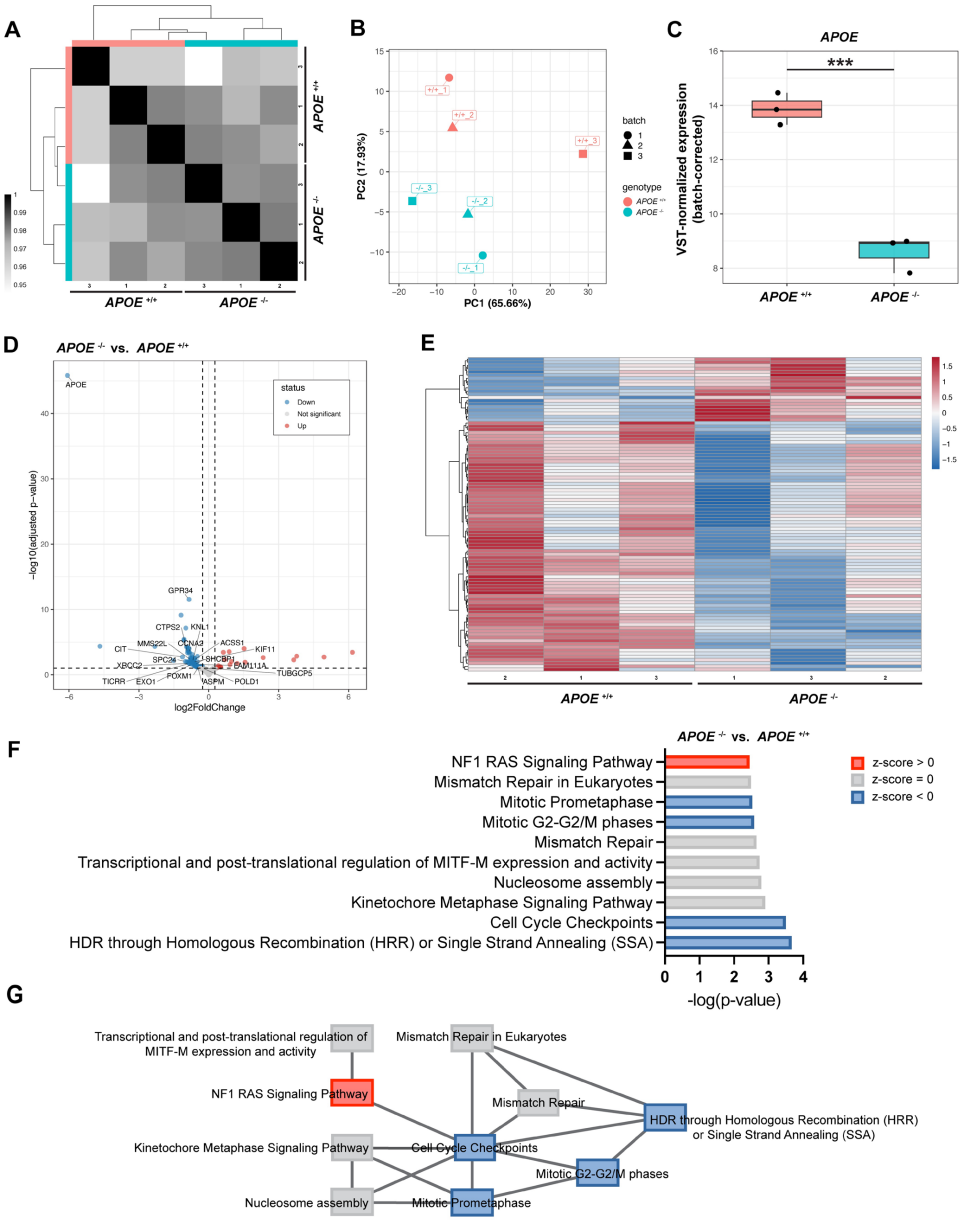
Transcriptomic profiling of *APOE* ^+/+^ and *APOE* ^−/−^ iMGLs by bulk RNA sequencing. (A) Sample‐to‐sample correlation matrix based on Spearman's rank coefficients, showing high reproducibility among biological replicates from independent differentiations (Sample size: *APOE* ^+/+^, *n* = 3; *APOE* ^−/−^, *n* = 3). (B) Principal component analysis (PCA) demonstrating distinct separation between *APOE* ^+/+^ and *APOE* ^−/−^ iMGLs. (C) Batch‐corrected, variance‐stabilised *APOE* expression levels confirming a significant decrease in *APOE* ^−/−^ iMGLs (***p < 0.001). (D) Volcano plot of differentially expressed genes (DEGs; adjusted *p* < 0.1, |log_2_ fold change| > 0.263, corresponding to ~1.2‐fold change). Labelled genes represent cell‐cycle‐related transcripts, except for *APOE* and *GPR34*. (E) Heatmap of DEGs shown in the volcano plot. (F) Canonical pathway analysis of DEGs using Ingenuity Pathway Analysis (IPA), showing the top 10 enriched pathways in *APOE* ^−/−^ iMGLs compared with *APOE* ^+/+^ iMGLs. Bars indicate pathway significance (−log(*p*‐value)), with colours reflecting predicted activation states (red, activated; blue, inhibited; grey, no predicted activation). (G) Pathway overlap network illustrating functional relationships among the enriched canonical pathways identified in panel F, based on shared DEGs. Node colours correspond to predicted activation states.

Sample‐to‐sample correlation coefficients (*p* = 0.95–1.00) indicated strong overall similarity across all samples (Figure 3A). The *APOE* ^+/+^ and *APOE* ^−/−^ samples also showed highly correlated global expression profiles, suggesting that the two genotypes exhibited minimal overall transcriptomic variation. PCA demonstrated distinct clustering of *APOE* ^+/+^ and *APOE* ^−/−^ iMGLs along PC1, which accounted for 65.66% of the total variance (Figure 3B). While the *APOE* ^+/+^ sample from batch 3 exhibited slightly lower pairwise correlation coefficients compared with other replicates and appeared modestly displaced along PC1, both correlation (*p* > 0.95) and clustering analyses indicated that it remained within the expected range of biological variation and was therefore not considered a technical outlier. Consistent with successful gene disruption, *APOE* transcript levels were significantly reduced in *APOE* ^−/−^ iMGLs compared with parental controls (Figure 3C).

Differential expression analysis identified a distinct transcriptional signature driven by *APOE* deletion (Figure 3D,E). A total of 42 upregulated and 84 downregulated genes were significantly altered (adjusted *p* < 0.1, |Fold change| > 1.2), which represents a relatively small number of transcriptional changes. Notably, several DEGs, such as *FOXM1*, *CCNA2* and *KIF11*, were involved in cell‐cycle regulation. In line with the qPCR results (Figure 1G), RNA‐seq analysis also revealed reduced expression of *GPR34* in *APOE* ^−/−^ iMGLs compared with *APOE* ^+/+^ controls (Figure 3D).

To gain functional insights, canonical pathway analysis was performed in IPA using the DEG list as input. The top 10 pathways ranked by *p*‐value (Figure 3F) revealed that several cell cycle–related pathways, such as Cell Cycle Checkpoints, Mitotic Prometaphase and Mitotic G_2_–G_2_/M Phases, were significantly downregulated (negative *z*‐scores). In addition, several DNA repair and chromatin organisation pathways, including Mismatch Repair, Homology‐Directed Repair and Nucleosome Assembly, were enriched without a defined activation direction (*z*‐score ≈ 0) or were predicted to be inhibited. Among the top‐ranked pathways, NF1 RAS Signalling was the only pathway predicted to be activated (positive *z*‐score). Neurofibromin (NF1), a Ras GTPase‐activating protein, negatively regulates Ras signalling and its downstream cascades, including the PI3K/Akt/mTOR pathway, which together control diverse cellular processes such as cell survival, proliferation and metabolism [29]. Its predicted activation in *APOE* ^−/−^ iMGLs thus also suggests a reduction in proliferative capacity, consistent with the overall downregulation of cell cycle–related pathways. To further explore functional connectivity among these pathways, pathway overlap analysis was performed to visualise their relationships based on shared DEGs. This analysis revealed that the Cell Cycle Checkpoints pathway served as a central node linking multiple dysregulated pathways (Figure 3G), suggesting that cell‐cycle dysregulation represents a major transcriptional consequence of *APOE* deletion in iMGLs.

In contrast to the pronounced lipid droplet accumulation phenotype in *APOE* ^−/−^ iMGLs (Figure 2E,F), unexpectedly, differential expression analysis showed no major alterations in canonical lipid metabolism genes such as *ABCA1*, *ACSL1* and *FASN*. To detect subtle transcriptional differences, we examined curated gene sets related to lipid metabolism from the Reactome, Gene Ontology Biological Process (GOBP) and Hallmark databases (Figure S3A). Notably, several genes involved in cholesterol biosynthesis, including *SQLE*, *HSD17B7* and *MVK*, showed mild upregulation in *APOE* ^−/−^ iMGLs, whereas lipid‐degrading enzymes including *PNPLA4* and *ASAH1* exhibited a trend towards reduced expression. The partial activation of these biosynthetic genes may expand the pool of free cholesterol available for esterification via acetyl‐CoA acetyltransferase 1 (ACAT1) and its subsequent deposition as cholesteryl esters within lipid droplets, consistent with the cholesteryl ester accumulation reported in *Apoe* ^−/−^ mouse microglia [28]. Although these transcriptional shifts did not reach statistical significance, the pattern was reproducible across independent replicates and suggested a coordinated attenuation of lipid catabolic programmes accompanied by partial activation of cholesterol storage genes, indicative of altered lipid handling in *APOE* ^−/−^ iMGLs. Together, these results indicate that while ApoE deficiency induces only modest global transcriptomic changes, it selectively downregulates cell cycle–associated genes and suggests a trend towards altered lipid homeostasis, reflecting a subtle imbalance between lipid catabolism and cholesterol storage in human *APOE* ^−/−^ iMGLs.

### 3.4 Proliferation Is Suppressed Under ApoE Deficiency in Human Microglia

**FIGURE 4:**
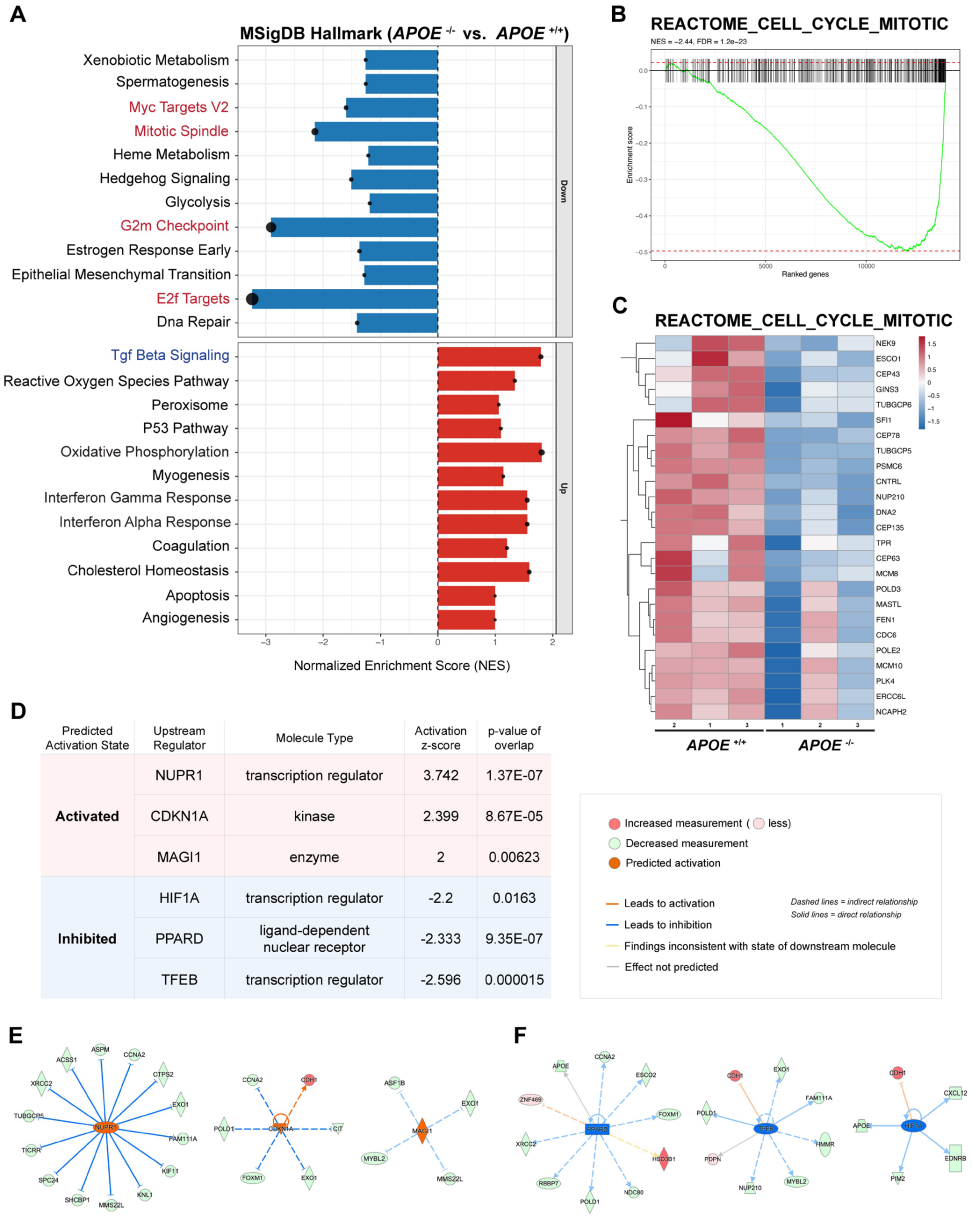
Decreased cell cycle–associated pathways and upstream regulators identified in *APOE* ^−/−^ iMGLs. (A) Fast Gene Set Enrichment Analysis (fGSEA; *APOE* ^−/−^ vs. *APOE* ^+/+^ iMGLs) highlighting downregulated cell cycle/proliferation pathways (red) and previously reported pathways (blue). Gene sets were obtained from the MSigDB Hallmark collection. (B) fGSEA enrichment plot for REACTOME_CELL_CYCLE_MITOTIC, showing downregulation in *APOE* ^−/−^ iMGLs. (C) Heatmap of leading‐edge genes contributing to the enrichment score, demonstrating reduced expression of cell cycle–associated genes within the REACTOME_CELL_CYCLE_MITOTIC gene set. (D) Top three activated and inhibited upstream regulators predicted by IPA, presented with activation *z*‐scores and *p*‐values of overlap. (E) Downstream target genes regulated by each of the activated upstream regulators shown in panel D. (F) Downstream target genes regulated by each of the inhibited upstream regulators shown in panel D.

Differential expression analysis revealed that ApoE deficiency profoundly altered the expression of genes associated with cell‐cycle regulation in iMGLs. However, given the relatively small number of DEGs, we considered the possibility that some pathways might be underrepresented in the DEG‐based analysis. To capture more subtle yet coordinated transcriptional changes, we therefore performed fast GSEA (fGSEA) using the entire ranked gene list, independent of DEG thresholds. This analysis uncovered additional pathways that were not detected by the over‐representation–based canonical pathway analysis. Consistent with previous observations in mouse *Apoe* ^−/−^ models [30], our analysis demonstrated enrichment of the TGF‐β signalling pathway in human *APOE* ^−/−^ iMGLs (Figure 4A). Moreover, cholesterol homeostasis pathways, which were not identified in the DEG‐based analysis, were positively enriched in ApoE‐deficient cells. Consistent with the canonical pathway analysis, fGSEA also revealed strong negative enrichment of multiple cell‐cycle regulation/proliferation‐associated gene sets, including HALLMARK_E2F_TARGETS, HALLMARK_G2M_CHECKPOINT and REACTOME_CELL_CYCLE_MITOTIC (Figure 4A, Figure S3B). The enrichment plots and leading‐edge heatmaps highlighted a coordinated downregulation of cell‐cycle regulators such as *PLK4*, *CDC6* and *MCM10*, pointing to a potential cell‐cycle arrest in *APOE* ^−/−^ iMGLs relative to *APOE* ^+/+^ controls (Figure 4B,C, Figure S3C). These results indicate that loss of ApoE may compromise the proliferative capacity of human microglia.

To explore potential regulators of these transcriptomic shifts in *APOE* ^−/−^ iMGLs, we next performed upstream regulator analysis. This analysis identified predicted activation of transcriptional regulators associated with stress responses and cell‐cycle control, and inhibition of transcription factors involved in metabolic and lysosomal regulation, including TFEB and PPARD (Figure 4D–F). Among these, the strongest predicted upstream regulator was NUPR1, a stress‐responsive transcription factor known to induce *CDKN1A* (p21) expression [31]. Upregulation of p21, a cyclin‐dependent kinase (CDK) inhibitor that was also identified as the third strongest upstream regulator in our analysis, suppresses CDK2–cyclin E/A and CDK1–cyclin B activities, thereby blocking both the G_1_/S and G_2_/M transitions [32, 33], suggesting that the NUPR1–p21 axis is a possible pathway mediating the transcriptomic alterations observed in *APOE* ^−/−^ iMGLs.

**FIGURE 5:**
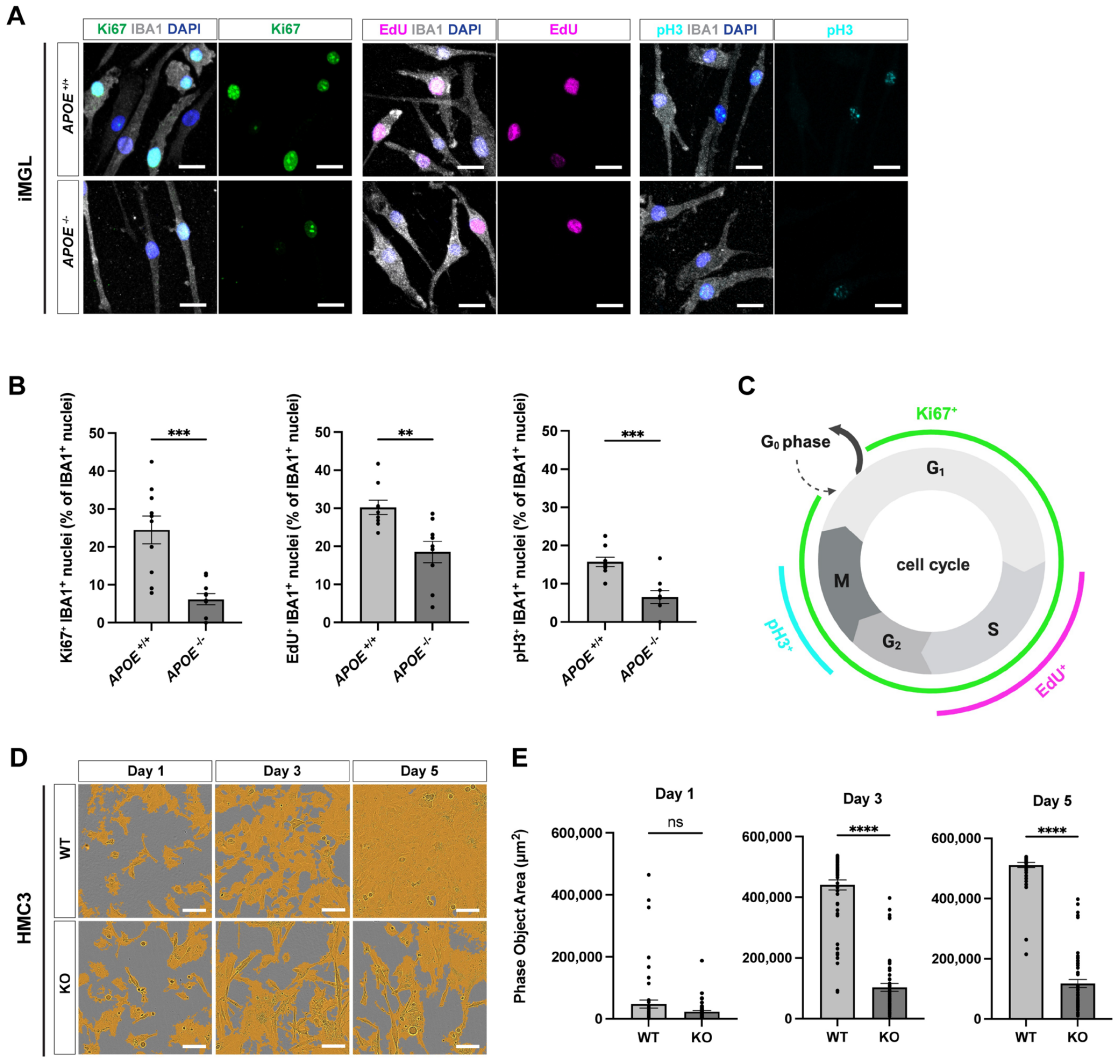
Experimental validation of reduced proliferative activity in *APOE* ^−/−^ human microglia. (A) Representative immunofluorescence images of Ki67 (green), EdU (magenta), phosphorylated histone H3 at serine 10 (pH 3; cyan) and IBA1 (grey) in iMGLs. Scale bars, 20 μm. (B) Quantification of Ki67^+^, EdU^+^ and pH 3^+^ cells among IBA1^+^ cells. EdU incorporation reflects cumulative labelling of cells that underwent DNA synthesis during the 24‐h labelling period. For Ki67, each data point represents one imaging field (2 fields per well, 1–2 wells per differentiation; total *n* = 10 fields from three independent differentiations). For EdU and pH 3, each data point represents one imaging field (1 field per well, 3 wells per differentiation; total *n* = 9 fields from three independent differentiations). Statistical significance was determined using an unpaired two‐tailed Student's *t*‐test (**p < 0.01; ***p < 0.001). (C) Schematic representation of cell‐cycle phases and the corresponding markers used in this study. Ki67 labelling (green) marks cells that have entered the cell cycle (G_1_, S, G_2_ and M phases) but is absent in quiescent G_0_ cells. EdU incorporation (magenta) identifies cells undergoing DNA synthesis during S phase. pH 3 labelling (cyan) marks cells in late G_2_ and M phases. Marker colours correspond to those used in the immunofluorescence staining shown in panel A. (D) Representative phase‐contrast images of the human microglial cell line HMC3 acquired by live‐cell imaging, showing changes in confluence over time (orange overlay indicates confluent area). Scale bars, 100 μm. (E) Comparison of phase‐object area (μm^2^) at days 1, 3 and 5 after cell seeding, showing no significant difference at day 1 but a significant reduction in *APOE* KO HMC3 cells at days 3 and 5, as determined by unpaired two‐tailed Student's *t*‐test (****p < 0.0001; *n* = 54 fields; nine fields from six wells per genotype; each dot represents one field).

To corroborate the transcriptomic evidence of reduced proliferative activity at the cellular level, we assessed proliferation by Ki67 immunostaining in iMGLs. A small fraction of the cells exhibited Ki67‐positive nuclei, indicating the presence of a subset of cells that had entered the cell cycle (Figure 5A). However, the proportion of Ki67‐positive cells was markedly reduced in *APOE* ^−/−^ iMGLs (Figure 5A–C), supporting an association between ApoE loss and reduced cell‐cycle entry in human microglial models. To further determine which stages of the cell cycle were affected, we next examined DNA synthesis and mitotic progression using EdU incorporation and phosphorylated histone H3 at serine 10 (pH 3) immunostaining, respectively. Consistent with the reduction in Ki67‐positive cells, *APOE* ^−/−^ iMGLs exhibited a significant decrease in the proportion of EdU‐positive cells, indicating impaired S‐phase entry (Figure 5A–C). In parallel, the fraction of pH 3‐positive cells, marking late G_2_/M‐phase and mitotic cells, was also markedly reduced in *APOE* ^−/−^ iMGLs (Figure 5A–C). To assess whether the proliferation defect associated with ApoE deficiency extends beyond iMGLs, we next examined proliferative activity in iPSCs and PMPs. In contrast to the pronounced reduction in proliferation markers observed in iMGLs, ApoE deficiency did not lead to a comparable decrease in Ki67‐, EdU‐, or pH 3‐positive cell populations in either iPSCs or PMPs (Figure S5A,B). These results suggest that the proliferative defect associated with ApoE loss becomes apparent upon microglial differentiation rather than at earlier developmental stages. Together, these findings demonstrate that ApoE deficiency leads to a microglia‐specific proliferative defect across multiple stages of the cell cycle, encompassing cell‐cycle entry, DNA replication and progression into mitosis, rather than selectively perturbing a single phase.

Given the inherently low proliferative capacity of iMGLs in vitro, as previously reported [34], and consistent with our observations based on Ki67‐positive cells (*APOE* ^+/+^: 26.8% ± 11.8%; *APOE* ^−/−^: 12.16% ± 10.1%), we employed a complementary system using the HMC3 human microglial cell line to further examine the mechanistic link between ApoE and proliferation. CRISPR‐Cas9–mediated KO of *APOE* in HMC3 cells yielded *APOE* KO HMC3, which retained expression of the microglial marker IBA1 and exhibited prominent BODIPY‐positive lipid droplets, recapitulating the phenotype observed in the iMGL models (Figure S4A–D). Time‐lapse live‐cell imaging over 120 h revealed a marked suppression of proliferation in *APOE* KO HMC3 cells compared with wild‐type (ε3/ε3) controls (Figure 5D, Figure S4E). Quantitative analysis confirmed significantly reduced confluence growth rates in ApoE‐deficient cells (Figure 5E, Figure S4F). Together, these findings demonstrate that ApoE loss intrinsically impairs microglial proliferative capacity, consistent with transcriptomic signatures of cell‐cycle repression.

**FIGURE 6:**
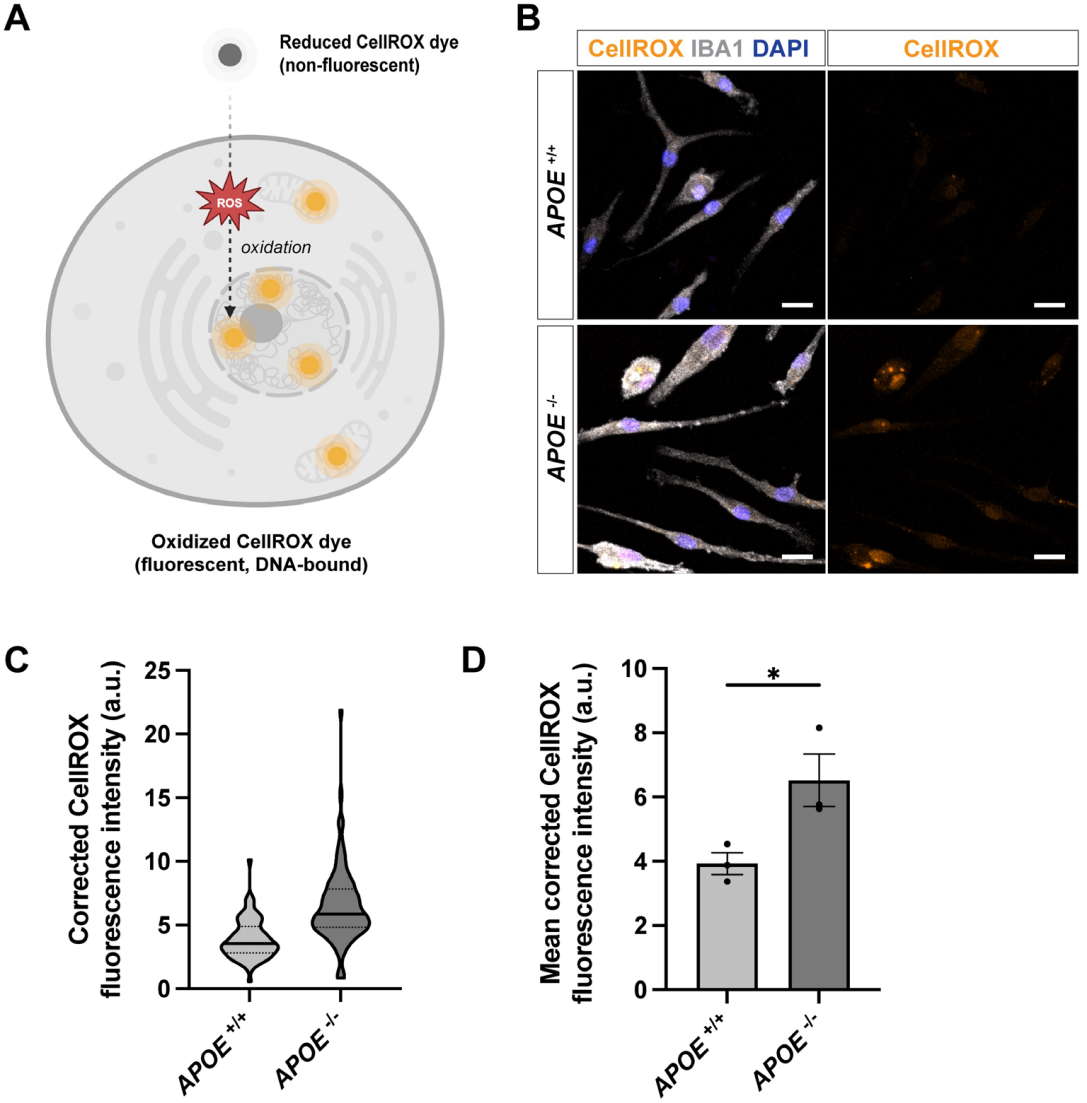
Increased intracellular oxidative stress in *APOE* ^−/−^ iMGLs assessed by CellROX Green. (A) Schematic illustration of the principle of CellROX Green–based oxidative stress detection. CellROX Green reagent is a cell‐permeant dye that remains non‐fluorescent in its reduced state. Under conditions of increased intracellular oxidative stress, reactive oxygen species (ROS) oxidise CellROX Green, converting it into a fluorescent, DNA‐bound form that accumulates in the nucleus and mitochondria. For clarity, the fluorescent signal is depicted in orange in the schematic and does not represent the actual emission wavelength. (B) Representative images of iMGLs immunostained for IBA1 (grey) and labelled with CellROX Green (orange). CellROX Green fluorescence was acquired using standard excitation/emission settings (485/520 nm) and is displayed in orange pseudocolor for visualisation purposes. (C) Violin plots showing the distribution of background‐subtracted CellROX mean fluorescence intensity at the single‐cell (IBA1‐defined ROI) level in iMGLs. (D) Comparison of mean corrected CellROX fluorescence intensity between genotypes after averaging values per well. Each dot represents one well (three wells per genotype). Statistical analysis was performed using an unpaired two‐tailed Student's *t*‐test (*p < 0.05).

Having established that ApoE deficiency intrinsically suppresses microglial proliferation, we next sought to explore potential pathways that might contribute to this phenotype. While the dominant transcriptomic signature was characterised by repression of cell‐cycle–related pathways, our fGSEA analysis also identified enrichment of HALLMARK_REACTIVE_OXYGEN_SPECIES pathways (Figure 4A), indicating altered redox homeostasis in *APOE* ^−/−^ iMGLs. To examine this observation at the cellular level, we assessed intracellular oxidative stress in iMGLs using CellROX, a fluorogenic probe for measuring oxidative stress in live cells (Figure 6A). This analysis revealed significantly elevated ROS signals in *APOE* ^−/−^ iMGLs compared with isogenic controls (Figure 6B–D). Together, these findings demonstrate that ApoE loss is associated with increased oxidative stress in human iMGLs, a cellular feature that may be linked to the reduced proliferative capacity observed in *APOE* ^−/−^ microglia.

## 4 Discussion

The present study aimed to investigate how the complete absence of ApoE affects human microglial function using an isogenic iPSC‐derived model. ApoE deficiency resulted in pronounced lipid droplet accumulation and enhanced NLRP3 inflammasome activation without affecting phagocytic activity, and it also markedly suppressed microglial proliferative capacity accompanied by downregulation of cell cycle–related genes (Figure S6A).

The lipid‐accumulation phenotypes observed in *APOE* ^‐/‐^ microglia are in agreement with previous reports [35], and are consistent with our transcriptomic signatures indicating enrichment patterns associated with altered lipid metabolism. In parallel with these lipid‐associated changes, we observed a significant increase in intracellular ROS levels in *APOE* ^−/−^ iMGLs. Given that intracellular lipid accumulation is known to promote ROS generation [36], which can activate the NLRP3 inflammasome [37], our findings suggest that lipid dysregulation caused by ApoE loss is accompanied by elevated oxidative stress that may facilitate NLRP3 inflammasome activation.

ApoE exists in three common isoforms in humans, ε2, ε3 and ε4, which differentially modulate AD risk [5]. Given the strong genetic association between *APOE* genotype and AD risk, extensive studies using human iPSC‐derived microglial models have demonstrated that *APOE* genotype influences not only lipid metabolism but also key aspects of AD pathophysiology by modulating innate immune signalling, lysosomal function and energy metabolism [9, 10, 11, 12, 13, 14, 15, 16, 17]. Mechanistically, the ε4 isoform has been proposed as exerting both loss‐of‐function (LoF) and gain‐of‐function (GoF) effects [38, 39, 40], yet their relative contribution remains incompletely defined [39]. Clarifying whether ε4‐associated perturbations primarily reflect LoF or GoF processes is therefore essential for understanding isoform‐specific mechanisms and rational therapeutic targeting. Within this framework, our model provides a comparative context that may help separate LoF‐driven alterations from ε4‐specific GoF signatures in human microglia. Consistent with this concept, ApoE deficiency produced lipid‐storage phenotypes resembling those reported in ε4 microglia [9, 17], in line with dysregulated lipid efflux pathways implicated in both contexts [16, 41]. Previous reports on ε4 microglial phagocytosis remain inconsistent, with some studies showing reduced phagocytic uptake of zymosan particles [9, 11], whereas others reported no significant differences under basal conditions [10]. Similarly, our data showed that ApoE loss did not directly affect basal engulfment mechanisms. Such discrepancies may reflect ε4‐specific GoF mechanisms, such as impaired lysosomal function [10, 16], or alternatively, experimental variations related to substrates or genetic background [10].

We further demonstrate that the loss of ApoE profoundly attenuates the proliferative capacity of human microglia, which, to our knowledge, has not been previously reported. Using iMGLs, we showed that human microglia can actively proliferate in vitro, and that *APOE* deletion diminishes this ability even under basal conditions. This finding reveals an important role of ApoE in maintaining microglial self‐renewal and suggests that ApoE can act beyond lipid transport, influencing the cell‐cycle machinery and potentially modulating AD pathology.

In AD brain, microglia surround amyloid plaques [42], which constitute a major pathological hallmark composed of aggregated amyloid beta (Aβ) proteins. Whether these plaque‐associated microglia are protective against, or detrimental to disease progression remains under debate and may depend on disease stage [43]. Early recruitment of microglia is thought to exert protective functions by forming a physical barrier and facilitating Aβ clearance [44], whereas chronic proliferation and activation are thought to exacerbate immune response and neuronal damage [45]. Notably, proliferating microglia increased in human postmortem AD brain and pharmacological inhibition of microglial proliferation alleviates cognitive functions without altering plaque burden in AD mouse models [46]. Moreover, clonal expansion of microglia has been observed in regions distant from plaques, followed by directed migration towards amyloid sites; blocking this clonal expansion reduces plaque burden [47]. These findings suggest that excessive or dysregulated microglial proliferation contributes to AD progression. Interestingly, under ApoE deficiency, the number of plaque‐associated microglia is reduced in parallel with attenuated amyloid‐induced tau pathology [48]. However, it remains unclear whether this reduction reflects impaired proliferation or reduced chemotaxis. ApoE has been shown to facilitate microglial migration towards amyloid plaques by VCAM1 signalling [49], yet its role in regulating microglia proliferation has remained largely unexplored. Our data provide initial evidence linking ApoE to microglial cell‐cycle regulation, and the consistent proliferation deficit observed across two distinct human microglial models suggests that this link may represent a universal feature of microglial biology, rather than one restricted to a particular differentiation stage or culture condition.

Previous in vivo studies have demonstrated that TREM2 signalling promotes local microglial expansion around amyloid plaques, whereas TREM2 deficiency or R47H LoF variants reduce microglial proliferation and clustering around plaques [50]. Because ApoE binds directly to TREM2 as an endogenous ligand [51], the absence of ApoE may blunt TREM2‐mediated proliferative responses. Indeed, treatment with TREM2‐agonistic antibodies restores proliferation in the AD mouse models expressing human *TREM2* R47H [52]. Yet, molecular simulations show that ApoE–TREM2 binding affinity follows the order ε4 > ε3 > ε2 [53], whereas both gene expression profiling [14] and proliferation marker analysis in demyelination models [54] indicate that microglial proliferation is highest in ε2 microglia, showing an opposite trend to TREM2 affinity. These findings suggest that binding affinity does not linearly translate to functional activation, implying that other signalling pathways might also be involved. Alternatively, chronic lipid stress may engage stress‐responsive regulators identified as upstream regulators in this study, such as NUPR1 and CDKN1A (p21), thereby contributing to cell‐cycle inhibition, consistent with the reduced proliferative activity observed in *APOE* ^−/−^ iMGLs. Together, these findings suggest that ApoE loss attenuates TREM2‐dependent microglial expansion and that lipid–cell‐cycle coupling may represent an additional regulatory layer governing microglial proliferation in AD.

ApoE is well recognised for its pivotal role in driving microglial state transitions under Aβ deposition or neurodegeneration, as demonstrated in both mouse and human microglia in vivo [30, 55]. However, our findings highlight that ApoE also applies significant modulatory influence under basal conditions, fine‐tuning microglial metabolic and proliferative programmes even in the absence of overt disease stimuli. In our data, ApoE deficiency resulted in relatively few differentially expressed genes, indicating that the complete loss of ApoE does not markedly alter the core identity of microglia. Yet, despite this limited transcriptomic impact, it selectively impairs specific functions such as lipid homeostasis and cell proliferation, suggesting that ApoE acts as a key regulator of microglial functional adaptability in response to environmental or metabolic cues across both steady‐state and disease contexts.

In this study, we observed transcriptional changes suggestive of altered TGF‐β signalling in *APOE* ^−/−^ iMGLs. fGSEA indicated enrichment of TGF‐β–related pathways, and qPCR analysis showed increased expression of TGF‐β signalling–associated genes *SMAD7* and *ID3* in *APOE* ^−/−^ iMGLs (Figure S3D). *SMAD7* is a well‐established TGF‐β–inducible negative feedback regulator [56], whereas *ID3* has been previously reported to be transcriptionally induced downstream of TGF‐β1 stimulation [57]. Although these results do not directly demonstrate activation of canonical TGF‐β signalling, such as SMAD2/3 phosphorylation or nuclear translocation, they are consistent with transcriptional modulation of TGF‐β–associated programmes. These findings are consistent with previous work by Krasemann et al. [30], who reported that *Tgfb1* is among the most derepressed genes in phagocytic microglia from *Apoe* ^−/−^ mice and proposed an inverse regulatory relationship between *Apoe* and TGF‐β–associated transcriptional programmes. While that study did not directly assess canonical TGF‐β signalling activity, the data support the notion that loss of ApoE is associated with derepression of TGF‐β–related transcriptional signatures in microglia. Importantly, the conservation of this transcriptional relationship between murine models and human iMGLs highlights the biological robustness and potential clinical relevance of this pathway. TGF‐β signalling is well established as a key regulator of microglial development and homeostasis, and has also been implicated in the control of microglial proliferation [23, 58, 59, 60]. Therefore, the observed transcriptional changes raise the possibility that altered TGF‐β–associated programmes may contribute to *APOE*‐dependent phenotypes in human iMGLs. However, whether this pathway plays a causal role downstream of ApoE deficiency will require further functional validation.

NUPR1, a stress‐inducible transcriptional regulator, is activated by various forms of cellular stress, including oxidative, endoplasmic reticulum and metabolic stress [61], and upregulates *CDKN1A* expression by directly binding to its promoter by forming a complex with p53 and p300 [31]. Previous studies have shown that lipid‐laden microglia exhibit elevated expression of genes associated with ROS production [36]. Consistent with these observations, we detected significantly increased intracellular ROS levels in *APOE* ^−/−^ iMGLs under basal conditions. Given that elevated oxidative stress is a known upstream activator of NUPR1 signalling, our observation of reduced proliferative activity in *APOE* ^−/−^ iMGLs may reflect a stress‐induced quiescent state driven by altered redox homeostasis. In this context, these findings implicate the NUPR1–p21 axis as a potential mechanistic link between metabolic perturbation, oxidative stress and cell‐cycle arrest, contributing to the anti‐proliferative phenotype observed under ApoE deficiency.

Importantly, p21 can mediate either reversible, stress‐induced quiescence or irreversible, senescence‐associated growth arrest. Thus, the identification of p21 as an upstream regulator in our data also raises the possibility of senescence‐associated cell‐cycle arrest. p21, a well‐characterised cyclin‐dependent kinase inhibitor that controls both the G_1_/S and G_2_/M checkpoints [32, 33], is upregulated during senescence and can mediate growth arrest via both p53‐dependent and ‐independent pathways [33, 62, 63]. Although our current results do not establish whether the reduced proliferation is directly related to cellular senescence, previous reports have suggested cell type–dependent associations between ApoE and senescence [64, 65]. However, in mouse microglia, *CDKN1A* induction suppresses proliferation without inducing canonical senescence markers [66], indicating that p21 alone is insufficient for defining a senescent state and that multiple markers need to be assessed [67]. Accordingly, determining whether the proliferation arrest observed in *APOE* ^−/−^ iMGLs reflects *bona fide* senescence or a metabolically driven quiescent state will be essential. Future mechanistic studies incorporating additional *APOE* genotypes, including ε2, ε4 and protective rare variants, together with targeted interrogation of TREM2 signalling, TGF‐β signalling–associated pathways, NUPR1–p21 axis, and p21‐associated senescence pathways in vitro and in vivo will provide crucial insights into how ApoE shapes microglial proliferation and AD progression.

This study has several limitations. First, our findings were derived from an in vitro model using iMGLs. While this system allows precise control of the genetic background and environmental factors, it does not fully recapitulate the multicellular complexity of the brain. Previous studies employing heterogeneous cell models or animal models have provided broader insights into the ApoE biology; however, those approaches often confound the cellular origin of ApoE, which can differ in post‐translational modifications and functional effects among neurons, astrocytes and microglia [68, 69, 70]. In contrast, our microglia‐specific system enables the investigation of cell‐autonomous roles of microglial ApoE, representing a unique strength of this work. Second, because the iPSCs were derived from a sporadic AD patient, the results may not be universally generalizable to all genetic or environmental backgrounds. The differences in proliferation‐related gene expression observed here, but not in previous studies [14], may reflect not only model‐dependent factors and environmental influences, but also donor‐specific variations. Finally, findings derived from iMGLs in a simplified in vitro context may not fully capture the context‐dependent regulation of proliferation observed in vivo [71]. Even within the brain, microglial proliferative activity is often confined to a limited subset or restricted to specific regions [72]. Thus, future studies using xenotransplantation models and single‐cell RNA sequencing will be crucial for elucidating how ApoE regulates microglial proliferation in a context‐dependent manner, thereby extending the conclusions drawn from this study.

## Author Contributions

**Dayoung Kim:** conceptualisation, methodology, investigation, formal analysis, writing – original draft, visualisation. **Takayuki Kondo:** conceptualisation, writing – review and editing, supervision, funding acquisition. **Keiko Imamura:** conceptualisation, writing – review and editing. **Kayoko Tsukita:** investigation. **Ayako Nagahashi:** investigation. **Tomoki Sakasai:** investigation. **Haruhisa Inoue:** conceptualisation, resources, writing – review and editing, supervision, project administration, funding acquisition.

## Ethics Statement

Generation and use of human iPSCs was approved by the Ethics Committee of the Department of Medicine and Graduate School of Medicine, Kyoto University (approved no. R0091 and G259). All experiments were conducted in compliance with the approved guidelines.

## Conflicts of Interest

The authors declare no conflicts of interest.

## Supporting information


**Figure S1:** Off‐target analysis of CRISPR‐Cas9‐mediated *APOE* knockout (KO).
**Figure S2:** Principal component analysis (PCA) of bulk RNA‐seq samples before batch correction.
**Figure S3:** Fast gene set enrichment analysis (fGSEA) of transcriptomic changes in *APOE* ^−/−^ iMGLs.
**Figure S4:** Validation of *APOE* knockout (KO) in HMC3 cells and characterisation of lipid and proliferative phenotypes that overlap with those observed in iMGLs.
**Figure S5:** Proliferation marker analysis in NANOG^+^ iPSCs and CD43^+^ primitive macrophage precursors (PMPs).
**Figure S6:** Graphical abstract of the present study.

## Data Availability

Data are available from the corresponding author upon reasonable request.
