# Supplementary material for: Apolipoprotein E Deficiency Impairs Human Microglial Proliferation Accompanied by Elevated Cellular Oxidative Stress
Source: J Cell Mol Med. 2026 Mar 20;30(6):e71074. doi: 10.1111/jcmm.71074 (PMC13097410; doi:10.1111/jcmm.71074)

Supplementary Figure 1. Off-target analysis of CRISPR-Cas9-mediated *APOE* knockout (KO).

(A) Overview of *in silico*–predicted off-target sites and validation results.

(B) Selected predicted off-target loci (OT1, chr9:126,460,257; OT2, chr5:138,964,879; and OT4, chr7:70,646,927; hg38) were analyzed by Sanger sequencing. No insertions or deletions were detected at the examined sites.

Supplementary Figure 2. Principal component analysis (PCA) of bulk RNA-seq samples before batch correction.

(A) Clustering was primarily driven by batch rather than genotype when comparing *APOE*^+/+^ and *APOE*^-/-^ iPSC-derived microglia-like cells (iMGLs) (Sample size: *APOE*^+/+^, *n* = 3; *APOE*^-/-^, *n* = 3).

Supplementary Figure 3. Fast gene set enrichment analysis (fGSEA) of transcriptomic changes in *APOE*^-/-^ iMGLs.

(A) Heatmap showing differential expression patterns of lipid metabolism–related genes curated from Hallmark, Gene Ontology Biological Process (GOBP), and Reactome databases between *APOE^+/+^* and *APOE*^-/-^ iMGLs.

(B) fGSEA (*APOE*^-/-^ vs. *APOE^+/+^* iMGLs) result using MSigDB Reactome gene sets identifying enriched pathways associated with cell-cycle regulation.

(C) fGSEA enrichment plot of for HALLMARK_E2F_TARGETS showing downregulation in *APOE*^-/-^ iMGLs.

(D) Selected genes contributing to the leading edge of the TGF-β signaling–associated gene set identified by fGSEA analysis were validated by quantitative PCR. Relative expression levels were compared between *APOE*^+/+^ and *APOE*^-/-^ iMGLs. Data are presented as mean ± SEM (*n* = 3 per genotype). Statistical significance was determined using an unpaired two-tailed Student’s *t*-test (** *p* < 0.01).

Supplementary Figure 4. Validation of *APOE* knockout (KO) in HMC3 cells and characterization of lipid and proliferative phenotypes that overlap with those observed in iMGLs.

(A) Schematic illustration of the CRISPR-Cas9 target site used to generate *APOE* KO HMC3 cells. The sequences below indicate the coding region of exon 2, with the underlined sequence corresponding to the edited region confirmed in panel C.

(B) Validation of *APOE* KO by Sanger sequencing demonstrating a stop-codon insertion, and by capillary western blot analysis.

(C) Representative fluorescence images of BODIPY (green), which labels neutral lipids, together with IBA1 (gray), showing lipid droplet accumulation in *APOE* KO HMC3 cells. Scale bars, 50 μm.

(D) Quantification of BODIPY⁺ area normalized to IBA1^+^ area (*n* = 6 fields; two fields from three wells). Statistical significance was determined using unpaired two-tailed Student’s *t*-test (* *p* < 0.05).

(E) Experimental timeline of live-cell imaging performed for 120 h after seeding HMC3 cells. Phase-contrast images were acquired at 4-h intervals.

(F) Quantification of phase-object confluence (%) over 120 h, showing slower growth kinetics in *APOE* KO HMC3 cells compared with wild-type (*n* = 54 fields; nine fields from six wells per genotype).

Supplementary Figure 5. Proliferation marker analysis in NANOG⁺ iPSCs and CD43⁺ primitive macrophage precursors (PMPs).

(A) Representative immunofluorescence images of Ki67, EdU, and phosphorylated histone H3 at serine 10 (pH3; cyan) co-stained with NANOG (gray) in iPSCs and with CD43 (gray) in PMPs. Scale bars, 20 μm.

(B) Quantification of Ki67⁺, EdU⁺, and pH3⁺ cells in NANOG⁺ iPSCs and CD43⁺ PMPs. EdU incorporation reflects cumulative labeling of cells that underwent DNA synthesis during a 1-h pulse. Each data point represents one imaging field (one field per well; three wells per genotype). Statistical significance was assessed using an unpaired two-tailed Student’s *t*-test.

Supplementary Figure 6. Graphical abstract of the present study.

A. iPSCs (*APOE* ε3/ε3; *APOE*^+/+^) were genetically engineered to generate *APOE* KO lines and subsequently differentiated into iMGLs. *APOE*^-/-^ iMGLs exhibited pronounced lipid droplet accumulation, elevated oxidative stress, enhanced NLRP3 inflammasome activation, and a marked reduction in proliferative capacity.

Supplementary Figure 1


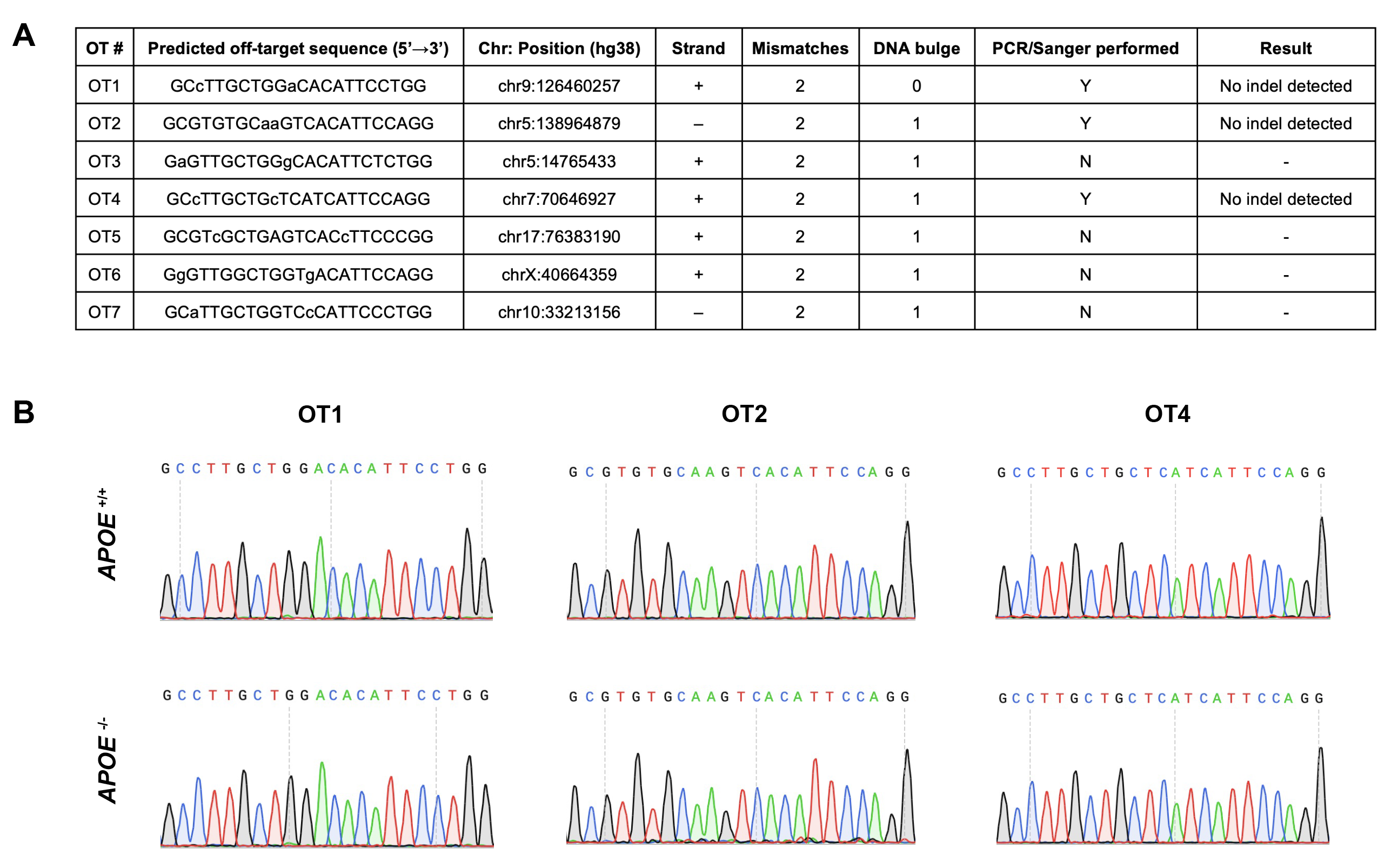


Supplementary Figure 2


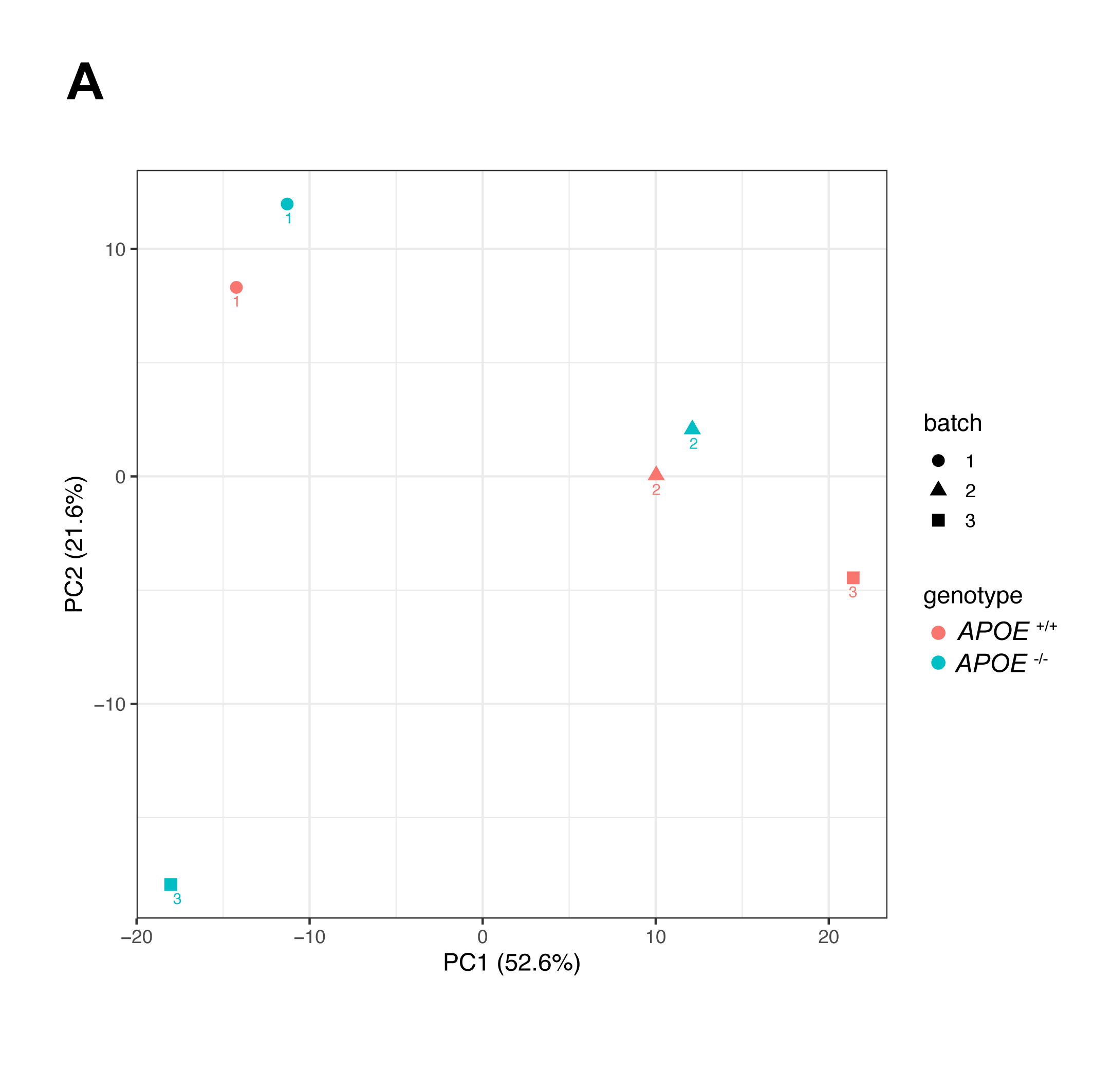


Supplementary Figure 3


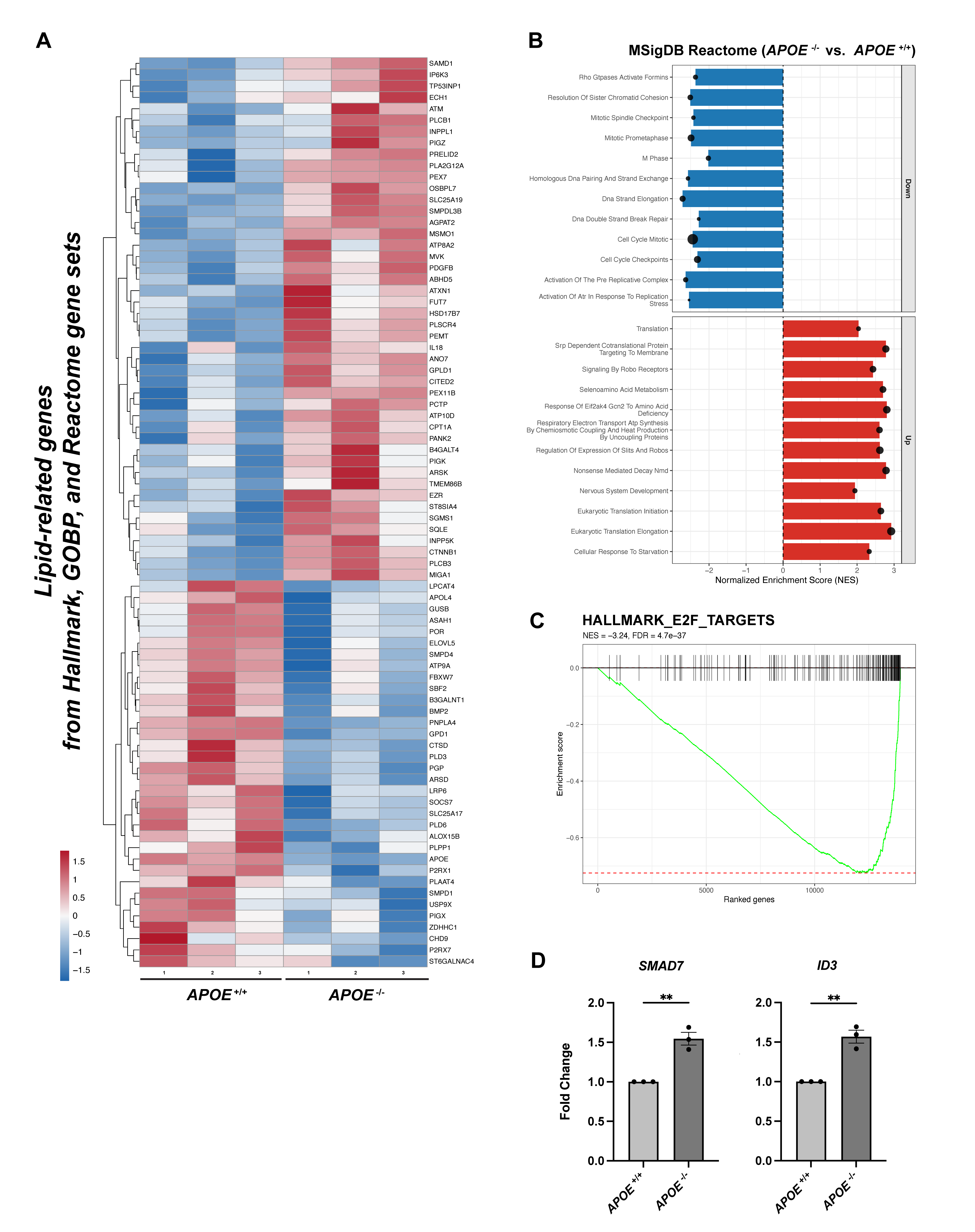


Supplementary Figure 4


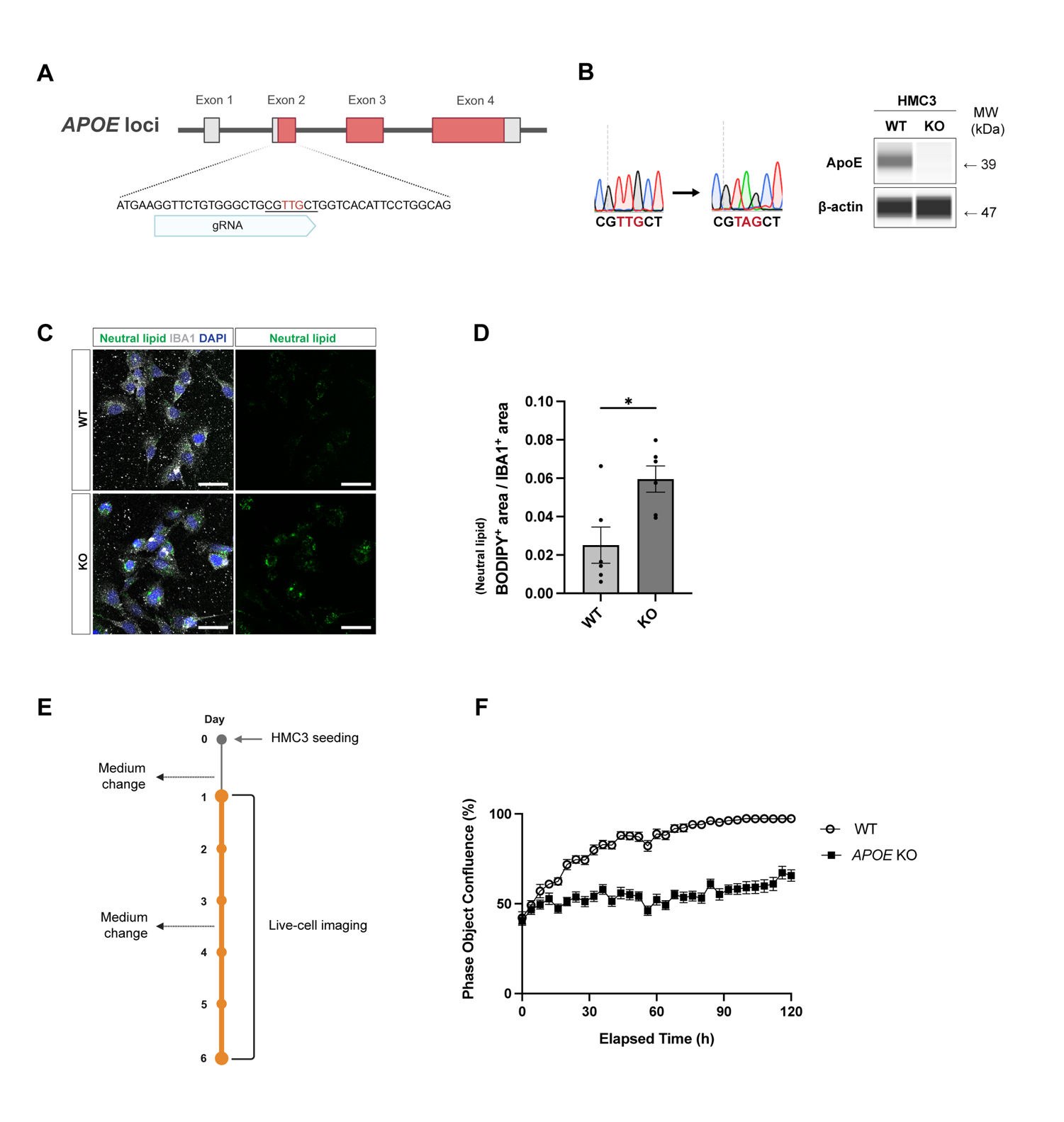


Supplementary Figure 5


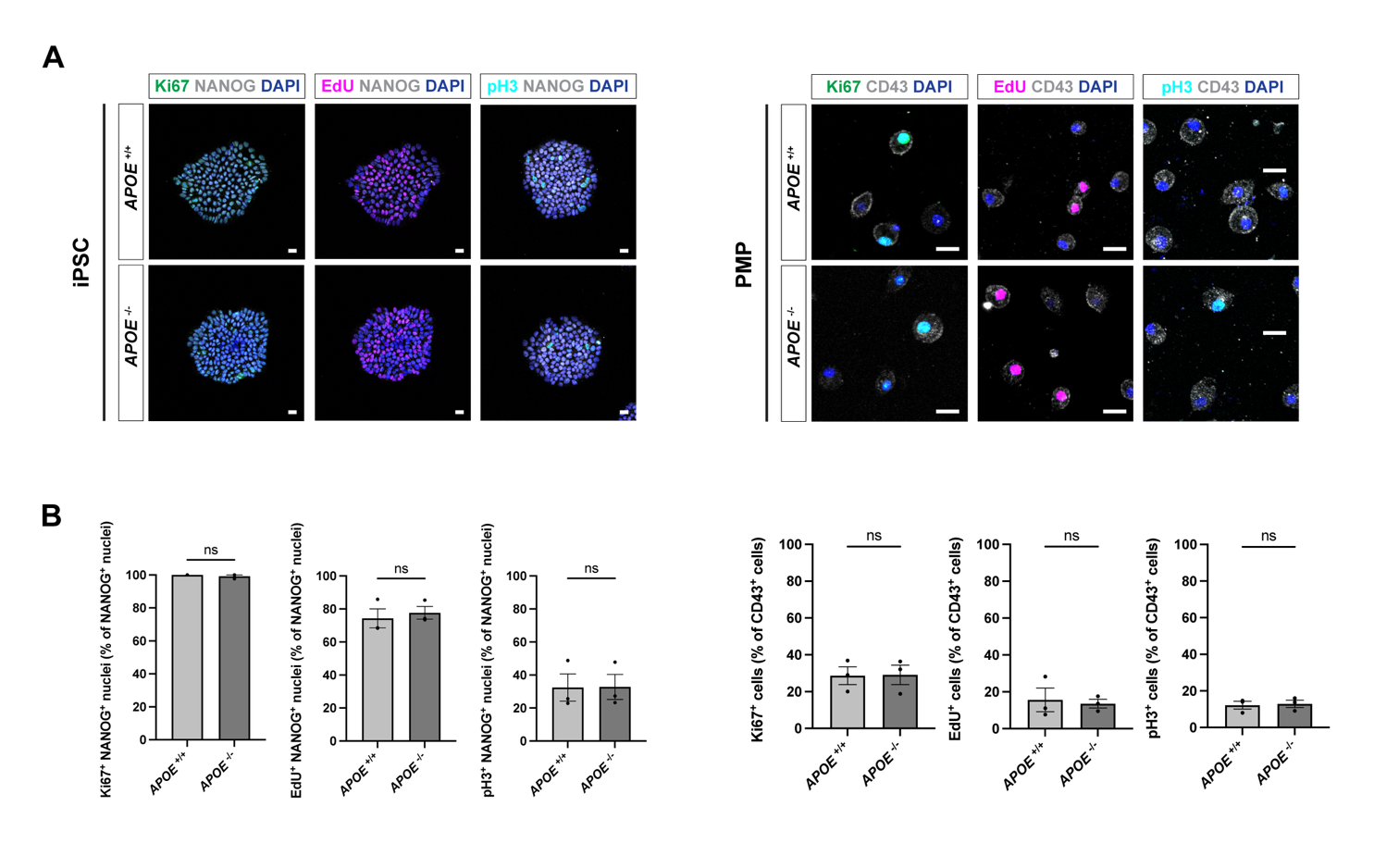


Supplementary Figure 6


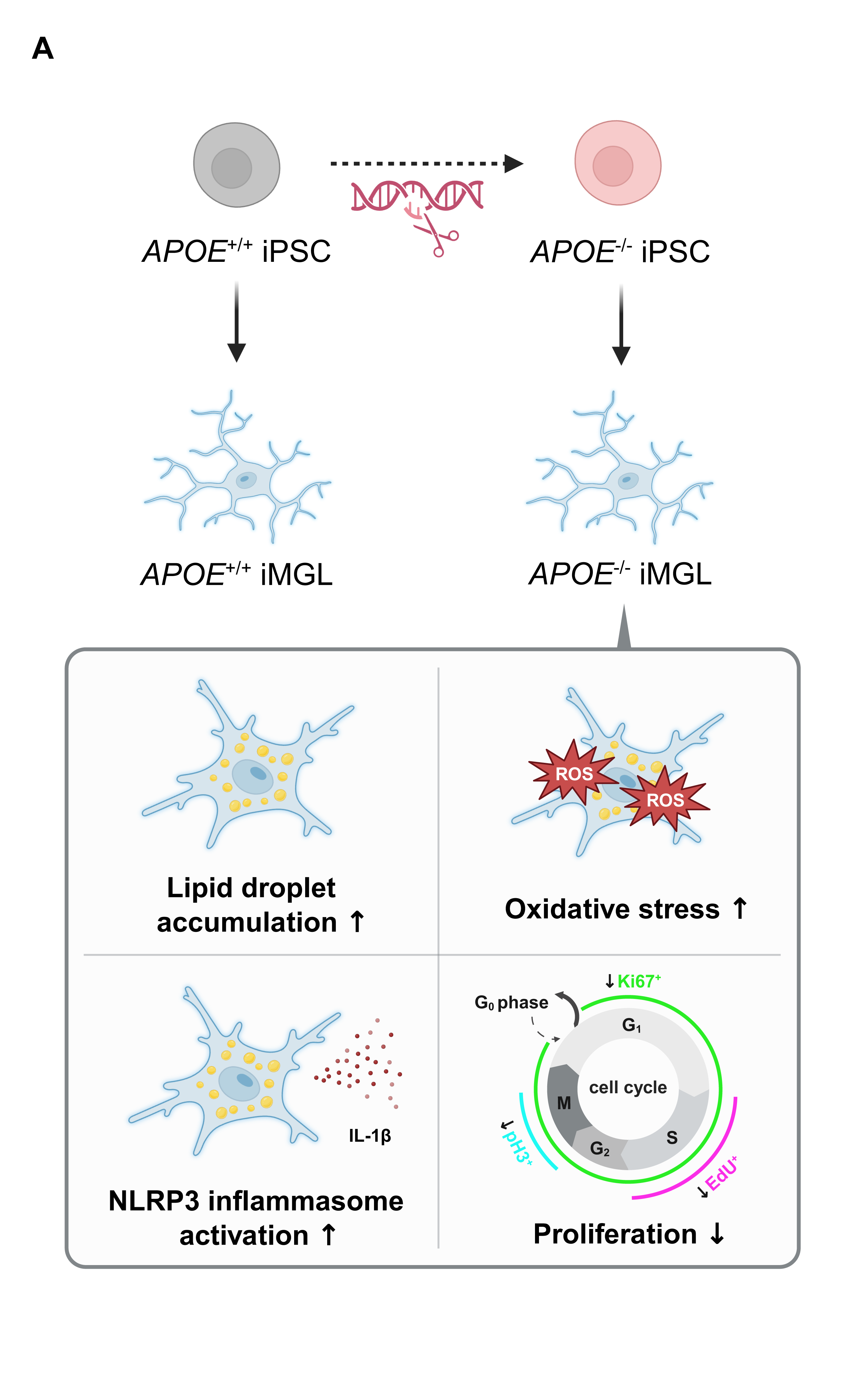

Supplement: Supplementary file 1 — Figure S1: Off‐target analysis of CRISPR‐Cas9‐mediated APOE knockout (KO). Figure S2: Principal component analysis (PCA) of bulk RNA‐seq samples before batch correction. Figure S3: Fast gene set enrichment analysis (fGSEA) of transcriptomic changes in APOE −/− iMGLs. Figure S4: Validation of APOE knockout (KO) in HMC3 cells and characterisation of lipid and proliferative phenotypes that overlap with those observed in iMGLs. Figure S5: Proliferation marker analysis in NANOG+ iPSCs and CD43+ primitive macrophage precursors (PMPs). Figure S6: Graphical abstract of the present study. [file JCMM-30-e71074-s001.docx]
